# Linear response of a superfluid Fermi gas inside its pair-breaking continuum

**DOI:** 10.1038/s41598-020-65371-9

**Published:** 2020-08-14

**Authors:** H. Kurkjian, J. Tempere, S. N. Klimin

**Affiliations:** 0000 0001 0790 3681 grid.5284.b TQC, Universiteit Antwerpen, Universiteitsplein 1, B-2610 Antwerp, Belgium

**Keywords:** Ultracold gases, Superconducting properties and materials, Bose-Einstein condensates

## Abstract

We study the signatures of the collective modes of a superfluid Fermi gas in its linear response functions for the order-parameter and density fluctuations in the Random Phase Approximation (RPA). We show that a resonance associated to the Popov-Andrianov (or sometimes “Higgs”) mode is visible inside the pair-breaking continuum at all values of the wavevector *q*, not only in the (order-parameter) modulus-modulus response function but also in the modulus-density and density-density responses. At nonzero temperature, the resonance survives in the presence of thermally broken pairs even until the vicinity of the critical temperature *T*_*c*_, and coexists with both the Anderson-Bogoliubov modes at temperatures comparable to the gap Δ and with the low-velocity phononic mode predicted by RPA near *T*_*c*_. The existence of a Popov-Andrianov-“Higgs” resonance is thus a robust, generic feature of the high-energy phenomenology of pair-condensed Fermi gases, and should be accessible to state-of-the-art cold atom experiments.

## Introduction

A primary way to probe the collective mode spectrum of a many-body system is by measuring the response functions of its macroscopic observables such as its density, or, in the case of a condensed system, its order parameter. These response functions can be measured by driving the system at a given wavenumber $q$ and varying the drive frequency $\omega$. In the theoretical case where the collective mode is undamped, one expects a infinitely narrow resonance (a Dirac peak) when $\omega$ coincides with the collective mode frequency ${\omega }_{{\bf{q}}}$. However, in most systems, collective modes are coupled to one or several continua of excitations, for example by intrinsic couplings to other elementary excitations. The system response in this case is less abrupt: the response functions are nonzero at all frequencies $\omega$ belonging to the continuum and the Dirac peak of the collective mode is replaced, in the favorable cases, by a broadened resonance. Theoretically, this damped resonance can be related to the existence of a pole in the analytic continuation of the response functions through their branch cuts associated to the continua^1–3^. Eventually, if the coupling to the continuum is very strong, the resonance may entirely disappear, such that only a slowly varying response remains visible inside the continuum.

Superfluid Fermi gases, which one can form by cooling down fermionic atoms prepared in two internal states $\uparrow /\downarrow$^4–13^, offer a striking example of this fundamental many-body phenomenon. This system of condensed pairs of $\uparrow /\downarrow$ fermions is described by 3 collective fields: the total density $\rho$ of particles and the phase and modulus of the order-parameter $\Delta$. In the general case, the fluctuations of those 3 fields are coupled and the collective modes have components on all of them. The system has also fermionic quasiparticles describing the breaking of pairs into unpaired fermions^14–17^, and two fermionic continua of quasiparticle biexcitations: a gapped quasiparticle-quasiparticle continuum and a gapless quasiparticle-quasihole continuum (to which the collective modes are coupled only at nonzero temperature). Since the coupling to these continua is not small in general, the collective mode spectrum can be obtained only after nonperturbative analytic continuations^18–21^. Performing an analytic continuation to study collective modes coupled to a continuum is a powerful heuristic tool: it is indispensable to interpret the shape of the response functions in terms of collective phenomena and to define precisely the spectrum of the collective branches. However, the poles found in the analytic continuation are not directly observable and one should always relate them to resonances which experiments can measure in the response functions.

Meanwhile, the experimental study of the collective modes of a superfluid Fermi gas is a very active field of research^8,13,22^, with a recent focus on the high-energy collective modes^23^ (at $\omega$ larger than the quasiparticle-quasiparticle continuum threshold at 2Δ) where a branch with quadratic dispersion^19,20^ is expected, reminiscent of the Higgs modes in high-energy physics^24^, superconductors^25–30^, superfluid fermionic Helium^31^ and nuclear matter^32,33^. This motivates us to discuss the observability, in the order-parameter and density response functions, of the collective modes (called Popov-Andrianov modes hereafter) predicted by refs. ^19,20,34^ based on the analytic structure of the functions continuated to imaginary frequencies. There are two major obstacles^24,28,35^ to the observation of the Popov-Andrianov-“Higgs” resonance in a conventional fermionic condensate. (*i*) So far the resonance has been clearly identified only in the modulus-modulus response function, whereas experiments (both in superconductors^24^ and ultracold Fermi gases^13,22^) usually excite or measure the density of the fermions. (*ii*) In a conventional fermionic condensate, where the resonance energy is above $2\Delta$ and the resonance broadened by its coupling to the pair-breaking continuum, it is generally not known whether a quality factor and spectral weight large enough to allow for an observation can be reached. Most studies then look for situations where the damping by the continuum is absent, as in Charged-Density-Wave superconductors^25,27–30^, inhomogeneous systems^36^ or superfluids in unconventional lattice geometries^35^. Here, we show that the resonance is observable in the density-density and density-modulus response functions at strong coupling even in a conventional fermionic condensate. In those density responses, the spectral weights of the resonance tends to zero with the wavevector $q$ while the quality factor decreases when $q$ increases. Nevertheless we could identify an intermediary regime ($q\approx \sqrt{2m\mu }$ at unitarity) where the resonance, and the characteristic quadratic dependence on $q$ of its peak frequency, should be resolvable from the continuum background in an ultracold Fermi gas.

We study the response functions in Anderson’s Random Phase Approximation (RPA)^37^. We use the formulation of ref. ^38^ in terms of bilinear quasiparticle operators that we generalize to nonzero temperature and to the presence of external drive fields. The RPA captures the coupling of the collective modes to the two fermionic continua (and the corresponding broadening of the resonances in the response functions) but neglects other couplings, in particular to the continua of two^39^, three^40,41^ or more collective excitations. We show that in this approximation, the density fluctuations are sensitive to the fluctuations of Δ, so that both modulus and phase collective modes are visible in the density response, but that the converse is not true. We give explicit expressions of each element of the response function matrix^1,42–44^, and show that they agree with path-integral based treatments^21,45^.

As the spectrum and response-function signatures of the low-energy collective modes is known in the RPA at zero^37,46,47^, nonzero temperature^38,48^ and near the critical temperature ${T}_{c}$^19,21,49^, we concentrate here on the high-energy ($\omega  > 2\Delta$) modes. At zero temperature, we show that the resonance of the Popov-Andrianov-“Higgs” mode is visible not only in the modulus-modulus response^20^ but also as a global extremum (in the region $\omega  > 2\Delta$) in the modulus-phase and modulus-density responses, and as a local extremum in the density-density response at strong coupling. As suggested by the analytic structure found in ref. ^34^, we show that the branch remains observable at large $q$ (in particular at $q\approx \sqrt{2m\mu }$ in the weak-coupling limit $\Delta \ll \mu$) with a quality factor below, but not much below unity.

At nonzero temperature, where the RPA captures the thermal population of the fermionic quasiparticle branches (and only of those branches) and describes the collective modes in the collisionless approximation, we show that the Popov-Andrianov resonance is not destroyed by the presence of thermally excited fermionic quasiparticles. On the contrary, the increase of temperature (which reduces Δ) favours the observability of the resonance in the density response functions by increasing the resonance spectral weight. The shape of the resonance is weakly affected by temperature, and for the order-parameter responses this shape is actually the same as at zero temperature for a slightly different interaction strength. Close to the critical temperature ${T}_{c}$, we show that collective mode in the pair-breaking continuum branch is not hidden by the low-velocity phononic branch^21^ as long as ${\hslash }^{2}{q}^{2}/m\lesssim {\Delta }^{2}/\mu$. This is in contrast with the Anderson-Bogoliubov branch, which disappears near ${T}_{c}$ according to the RPA.

Altogether our findings confirm the observability of the Popov-Andrianov-“Higgs” branch, which appears, after our study, as the strongest feature of the high-energy phenomenology of pair-condensed Fermi gases. It is observable in wide ranges of values of the interaction strength, exciting wavevector and temperature, and it is only weakly affected by the singularities caused in the response functions by the structure changes of the fermionic continuum. We are then optimistic about its observability, especially if the experiments can access one of the modulus response functions (modulus-modulus, modulus-phase or modulus-density). The modulus of the order-parameter can be excited by a Feshbach modulation of the scattering length^20,50^, after which the modulus-density response can be measured by absorption images as in refs. ^13,22^. Alternatively the density can be excited by a Bragg pulse^13^ or by shaking the confinement walls^22^, and the order-parameter modulus measured after a bosonization of the Cooper pairs. In the density-density response, it would be interesting to see if the peak observed in^13^ above 2Δ has the characteristic behavior of the Popov-Andrianov-“Higgs” mode, that is, a quadratic dependence on $q$ for both the peak frequency and its width.

## BCS Theory At Nonzero Temperature

To derive the matrix of linear response functions, we use the formalism of ref. ^38^, itself based on the RPA approach of Anderson^37^, and we generalize it to the presence of pairing and density exciting fields. We start by briefly recalling the formalism of BCS theory at nonzero temperature. In real and momentum space, the Hamiltonian of an isolated gas of fermions in two internal states $\sigma =\uparrow /\downarrow$ with $s$-wave contact interactions is given by

1 $$\begin{array}{l}\hat{H}={l}^{3}\,\sum _{{\bf{r}},\sigma =\uparrow /\downarrow }\,{\hat{\psi }}_{\sigma }^{\dagger }({\bf{r}})\left(\,-\frac{1}{2m}{\Delta }_{{\bf{r}}}-\mu \right){\hat{\psi }}_{\sigma }({\bf{r}})+\,{g}_{0}{l}^{3}\,\sum _{{\bf{r}}}\,{\hat{\psi }}_{\uparrow }^{\dagger }({\bf{r}}){\hat{\psi }}_{\downarrow }^{\dagger }({\bf{r}}){\hat{\psi }}_{\downarrow }({\bf{r}}){\hat{\psi }}_{\uparrow }({\bf{r}})\end{array}$$

2 $$=\sum \,_{{\bf{k}}\in {\mathcal{D}},\sigma =\uparrow /\downarrow }\,\left(\frac{{k}^{2}}{2m}-\mu \right){\hat{a}}_{{\bf{k}}\sigma }^{\dagger }{\hat{a}}_{{\bf{k}}\sigma }+\frac{{g}_{0}}{V}\,\sum _{{\bf{k}},{\bf{k}}{\prime} ,{\bf{q}}\in {\mathcal{D}}}\,{\hat{a}}_{{\bf{k}}{\prime} \uparrow }^{\dagger }{\hat{a}}_{-{\bf{k}}{\prime} -{\bf{q}}\downarrow }^{\dagger }{\hat{a}}_{-{\bf{k}}-{\bf{q}}\downarrow }{\hat{a}}_{{\bf{k}}\uparrow }\mathrm{}.$$

We use from now on the convention $\hslash ={k}_{{\rm{B}}}=1$. To introduce a momentum cutoff in a natural way, we discretize space into a cubic lattice of step $l$ and impose periodic boundary conditions (in a volume $V={L}^{3}$), which restrict the values of the wavevectors to ${\mathcal{D}}=\frac{2\pi }{L}{{\mathbb{Z}}}^{3}\cap {[-\pi /l,\pi /l]}^{3}$. The bare coupling constant ${g}_{0}$ is renormalized to reproduce the correct $s$-wave scattering length of the two-body problem:

3 $$\frac{1}{{g}_{0}}=\frac{m}{4\pi {\hslash }^{2}a}-{\int }_{{[-\pi /l,\pi /l]}^{3}}\,\frac{{d}^{3}k}{{(2\pi )}^{3}}\frac{m}{{k}^{2}}.$$

At the end of the calculation we take the lattice spacing $l$ to $0$, and thus ${g}_{0}$ tends to $0$ to compensate the divergence of the integral on the right-hand-side of (3).

BCS theory describes the equilibrium state at temperature $T$ by the Gaussian state:

4 $${\hat{\rho }}_{{\rm{BCS}}}(T)=\frac{\exp (-\,{\hat{H}}_{{\rm{BCS}}}/T)}{{\mathcal{Z}}}$$

where ${\mathcal{Z}}$ is the partition function and the BCS Hamiltonian ${H}_{{\rm{BCS}}}$ is obtained by treating the interactions in the mean-field approximation, *i*.*e*. by replacing the quartic interaction term ${g}_{0}{\hat{\psi }}_{\uparrow }^{\dagger }({\bf{r}}){\hat{\psi }}_{\downarrow }^{\dagger }({\bf{r}}){\hat{\psi }}_{\downarrow }({\bf{r}}){\hat{\psi }}_{\uparrow }({\bf{r}})$ in (2) by a quadratic one $\Delta ({\hat{\psi }}_{\uparrow }^{\dagger }({\bf{r}}){\hat{\psi }}_{\downarrow }^{\dagger }({\bf{r}})+{\rm{c}}.{\rm{c}}.)$, through the introduction of the self-consistent pairing-field

5 $$\Delta ={g}_{0}{\langle {\hat{\psi }}_{\uparrow }^{\dagger }({\bf{r}}){\hat{\psi }}_{\downarrow }^{\dagger }({\bf{r}})\rangle }_{T}\mathrm{}.$$

Here ${\langle \ldots \rangle }_{T}$ denotes the average in the thermal state ${\hat{\rho }}_{{\rm{BCS}}}(T)$. This quadratic Hamiltonian can be diagonalized easily into a Hamiltonian describing fermionic elementary excitations on top of a ground state energy ${E}_{0}$:

6 $${\hat{H}}_{{\rm{BCS}}}={E}_{0}+\sum _{{\bf{k}}\in {\mathcal{D}}}\,{\epsilon }_{{\bf{k}}}{\hat{\gamma }}_{{\bf{k}}\sigma }^{\dagger }{\hat{\gamma }}_{{\bf{k}}\sigma }\mathrm{}.$$

Here the eigenenergy of a fermionic excitation is

7 $${\epsilon }_{{\bf{k}}}=\sqrt{{\xi }_{{\bf{k}}}^{2}+{\Delta }^{2}}\,{\rm{with}}\,{\xi }_{{\bf{k}}}=\frac{{k}^{2}}{2m}-\mu \mathrm{}.$$

The fermionic quasiparticle operators ${\hat{\gamma }}_{{\bf{k}},\sigma }$ are obtain after a Bogoliubov rotation of the particle operators ${\hat{a}}_{{\bf{k}},\sigma }$ as in the zero temperature case:

8 $${\hat{\gamma }}_{{\bf{k}}\uparrow }={U}_{{\bf{k}}}{\hat{a}}_{{\bf{k}}\uparrow }+{V}_{{\bf{k}}}{\hat{a}}_{-{\bf{k}}\downarrow }^{\dagger }$$

9 $${\hat{\gamma }}_{-{\bf{k}}\downarrow }=-\,{V}_{{\bf{k}}}{\hat{a}}_{{\bf{k}}\uparrow }^{\dagger }+{U}_{{\bf{k}}}{\hat{a}}_{-{\bf{k}}\downarrow }$$

with the Bogoliubov coefficients *U*_**k**_ and *V*_**k**_:

10 $${U}_{{\bf{k}}}=\sqrt{1+\frac{{\xi }_{{\bf{k}}}}{{\epsilon }_{{\bf{k}}}}}\,{\rm{and}}\,{V}_{{\bf{k}}}=\sqrt{1-\frac{{\xi }_{{\bf{k}}}}{{\epsilon }_{{\bf{k}}}}}\mathrm{}.$$

The difference with the zero temperature case lies in the average values of the bilinear operators:

11 $$\langle {\hat{a}}_{{\bf{k}},\sigma }^{\dagger }{\hat{a}}_{{\bf{k}},\sigma }\rangle =({U}_{{\bf{k}}}^{2}-{V}_{{\bf{k}}}^{2}){f}_{{\bf{k}}}+{V}_{{\bf{k}}}^{2}$$

12 $$\langle {\hat{a}}_{-{\bf{k}},\downarrow }{\hat{a}}_{{\bf{k}},\uparrow }\rangle =-\,(1-2{f}_{{\bf{k}}}){U}_{{\bf{k}}}{V}_{{\bf{k}}},$$

which now depend on the Fermi-Dirac occupation number

13 $${f}_{{\bf{k}}}={\langle {\hat{\gamma }}_{{\bf{k}},\sigma }^{\dagger }{\hat{\gamma }}_{{\bf{k}},\sigma }\rangle }_{T}=\frac{1}{1+\exp \,{\epsilon }_{{\bf{k}}}/T}\mathrm{}.$$

This thermal population of the quasiparticle modes also affect the gap equation:

14 $$\Delta =-\,\frac{{g}_{0}}{{L}^{3}}\,\sum _{{\bf{k}}\in {\mathcal{D}}}\,{U}_{{\bf{k}}}{V}_{{\bf{k}}}\mathrm{(1}-2{f}_{{\bf{k}}}\mathrm{)}.$$

Thus, at nonzero temperature, BCS theory captures the effects due to the thermally excited fermionic quasiparticles (the broken pairs); it completely neglects that there are also thermally excited collective modes (some of which are gapless) which is a serious limitation, particularly at strong coupling.

## RPA Equations of Motion in Presence of Drive Fields

To study the linear response of the gas, we introduce, on top of the Hamiltonian (2) of the isolated gas, a quadratic Hamiltonian describing the experimental driving of the system:

15 $$\begin{array}{rcl}{\hat{H}}_{{\rm{drive}}} & = & {l}^{3}\,\sum _{{\bf{r}}}\,({u}_{\uparrow }({\bf{r}},t){\hat{\psi }}_{\uparrow }^{\dagger }({\bf{r}}){\hat{\psi }}_{\uparrow }({\bf{r}})+{u}_{\downarrow }({\bf{r}},t){\hat{\psi }}_{\downarrow }^{\dagger }({\bf{r}}){\hat{\psi }}_{\downarrow }({\bf{r}})\mathrm{}\\  &  & +\,[{\hat{\psi }}_{\uparrow }^{\dagger }({\bf{r}}){\hat{\psi }}_{\downarrow }^{\dagger }({\bf{r}})\phi ({\bf{r}},t)+{\rm{c}}.{\rm{c}}\mathrm{}.]).\end{array}$$

Here the fields ${u}_{\sigma }({\bf{r}})$, coupled to the density of spin $\sigma$ fermions, describe for instance a Bragg excitation of the gas^13^. The complex field $\phi ({\bf{r}})$ coupled to the quantum pairing field ${\hat{\psi }}_{\uparrow }^{\dagger }({\bf{r}}){\hat{\psi }}_{\downarrow }^{\dagger }({\bf{r}})$ can be imposed for instance by a Feshbach-modulation of the interaction strength^50^. An excitation coupled to the phase of ${\hat{\psi }}_{\uparrow }^{\dagger }({\bf{r}}){\hat{\psi }}_{\downarrow }^{\dagger }({\bf{r}})$ can be achieved using a time- and space-dependent Josephson junction as proposed in ref. ^21^. This drive Hamiltonian decomposes into a sum of Fourier components of the momentum ${\bf{q}}$ transferred to system:

16 $$\begin{array}{rcl}{\hat{H}}_{{\rm{drive}}} & = & \sum _{{\bf{q}}}\,{\hat{H}}_{{\rm{drive}}}({\bf{q}})\,{\rm{with}}\\ {\hat{H}}_{{\rm{drive}}}({\bf{q}}) & = & \sum _{{\bf{k}}}\,({u}_{\uparrow }(\,-\,{\bf{q}}){\hat{n}}_{{\bf{k}}}^{{\bf{q}}}+{u}_{\downarrow }(\,-\,{\bf{q}}){\hat{\bar{n}}}_{{\bf{k}}}^{{\bf{q}}})+\phi (\,-\,{\bf{q}})\,\sum _{{\bf{k}}}\,{\hat{\bar{d}}}_{{\bf{k}}}^{{\bf{q}}}+\bar{\phi }(\,-\,{\bf{q}})\,\sum _{{\bf{k}}}\,{\hat{d}}_{{\bf{k}}}^{{\bf{q}}}\mathrm{}.\end{array}$$

We use here a symmetrized version of Anderson’s notations^37^ for the bilinear fermion operators (see also chapter V. in ref. ^51^),

17 $$\begin{array}{ll}{\hat{n}}_{{\bf{k}}}^{{\bf{q}}}={\hat{a}}_{{\bf{k}}+{\bf{q}}\mathrm{/2}\uparrow }^{\dagger }{\hat{a}}_{{\bf{k}}-{\bf{q}}\mathrm{/2}\uparrow } & {\hat{\bar{n}}}_{{\bf{k}}}^{{\bf{q}}}={\hat{a}}_{-{\bf{k}}+{\bf{q}}\mathrm{/2}\downarrow }^{\dagger }{\hat{a}}_{-{\bf{k}}-{\bf{q}}\mathrm{/2}\downarrow }\\ {\hat{d}}_{{\bf{k}}}^{{\bf{q}}}={\hat{a}}_{-{\bf{k}}-{\bf{q}}\mathrm{/2}\downarrow }{\hat{a}}_{{\bf{k}}-{\bf{q}}\mathrm{/2}\uparrow } & {\hat{\bar{d}}}_{{\bf{k}}}^{{\bf{q}}}={\hat{a}}_{{\bf{k}}+{\bf{q}}\mathrm{/2}\uparrow }^{\dagger }{\hat{a}}_{-{\bf{k}}+{\bf{q}}\mathrm{/2}\downarrow }^{\dagger }\end{array}$$

and the Fourier transforms of the drive fields:

18 $${u}_{\sigma }({\bf{q}})=\frac{{l}^{3}}{V}\,\sum _{{\bf{r}}}\,{e}^{i{\bf{q}}\cdot {\bf{r}}}{u}_{\sigma }({\bf{r}})$$

19 $$\phi ({\bf{q}})=\frac{{l}^{3}}{V}\,\sum _{{\bf{r}}}\,{e}^{i{\bf{q}}\cdot {\bf{r}}}\phi ({\bf{r}})\,{\rm{and}}\,\bar{\phi }({\bf{q}})={\phi }^{\ast }(\,-\,{\bf{q}}\mathrm{)}.$$

In the framework of linear response theory, we seek the response of the system to first order in the fields $\phi$ and ${u}_{\sigma }$. We thus neglect the quantum fluctuations in the terms of the equations of motion deriving from ${\hat{H}}_{{\rm{drive}}}$:

20 $$[\hat{a}\hat{b},{\hat{H}}_{{\rm{drive}}}]\simeq {\langle [\hat{a}\hat{b},{\hat{H}}_{{\rm{drive}}}]\rangle }_{T},$$

where the average value ${\langle [\hat{a}\hat{b},{\hat{H}}_{{\rm{drive}}}]\rangle }_{T}$ is taken in the BCS equilibrium state at zero fields. The rest of the derivation is similar to what is explained in refs. ^38,51^: one writes the Heisenberg equations of motion for the bilinear fermionic operators (17) and linearizes them using incomplete Wick contractions (*i*.*e*. the replacement $\hat{a}\hat{b}\hat{c}\hat{d}\to \hat{a}\hat{b}{\langle \hat{c}\hat{d}\rangle }_{T}+{\langle \hat{a}\hat{b}\rangle }_{T}\hat{c}\hat{d}-\hat{a}\hat{c}{\langle \hat{b}\hat{d}\rangle }_{T}-{\langle \hat{a}\hat{c}\rangle }_{T}\hat{b}\hat{d}+\ldots$). The resulting equations of motion of the bilinear particle operators are given in Appendix A. We give here the equations of motion in their simplest form, which is in the quasiparticle basis. At the level of the bilinear operators, the Bogoliubov rotation (8) and (9) becomes:

21 $$(\begin{array}{c}{\hat{y}}_{{\bf{k}}}^{{\bf{q}}}\\ {\hat{h}}_{{\bf{k}}}^{{\bf{q}}}\\ {\hat{s}}_{{\bf{k}}}^{{\bf{q}}}\\ {\hat{m}}_{{\bf{k}}}^{{\bf{q}}}\end{array})\equiv (\begin{array}{c}{\hat{\gamma }}_{-{{\bf{k}}}_{+},\downarrow }{\hat{\gamma }}_{{{\bf{k}}}_{-},\uparrow }-{\hat{\gamma }}_{{{\bf{k}}}_{+},\uparrow }^{\dagger }{\hat{\gamma }}_{-{{\bf{k}}}_{-},\downarrow }^{\dagger }\\ {\hat{\gamma }}_{{{\bf{k}}}_{+}\uparrow }^{\dagger }{\hat{\gamma }}_{{{\bf{k}}}_{-}\uparrow }-{\hat{\gamma }}_{-{{\bf{k}}}_{+}\downarrow }^{\dagger }{\hat{\gamma }}_{-{{\bf{k}}}_{-}\downarrow }\\ {\hat{\gamma }}_{-{{\bf{k}}}_{+},\downarrow }{\hat{\gamma }}_{{{\bf{k}}}_{-},\uparrow }+{\hat{\gamma }}_{{{\bf{k}}}_{+},\uparrow }^{\dagger }{\hat{\gamma }}_{-{{\bf{k}}}_{-},\downarrow }^{\dagger }\\ {\hat{\gamma }}_{{{\bf{k}}}_{+}\uparrow }^{\dagger }{\hat{\gamma }}_{{{\bf{k}}}_{-}\uparrow }+{\hat{\gamma }}_{-{{\bf{k}}}_{+}\downarrow }^{\dagger }{\hat{\gamma }}_{-{{\bf{k}}}_{-}\downarrow }\end{array})=(\begin{array}{cccc}{W}_{{\bf{k}}{\bf{q}}}^{+} & {w}_{{\bf{k}}{\bf{q}}}^{-} & 0 & 0\\ -{w}_{{\bf{k}}{\bf{q}}}^{-} & {W}_{{\bf{k}}{\bf{q}}}^{+} & 0 & 0\\ 0 & 0 & {W}_{{\bf{k}}{\bf{q}}}^{-} & -{w}_{{\bf{k}}{\bf{q}}}^{+}\\ 0 & 0 & {w}_{{\bf{k}}{\bf{q}}}^{+} & {W}_{{\bf{k}}{\bf{q}}}^{-}\end{array})(\begin{array}{c}{\hat{d}}_{{\bf{k}}}^{{\bf{q}}}-{\hat{\bar{d}}}_{{\bf{k}}}^{{\bf{q}}}\\ {\hat{n}}_{{\bf{k}}}^{{\bf{q}}}-{\hat{\bar{n}}}_{{\bf{k}}}^{{\bf{q}}}\\ \delta ({\hat{d}}_{{\bf{k}}}^{{\bf{q}}}+{\hat{\bar{d}}}_{{\bf{k}}}^{{\bf{q}}})\\ \delta ({\hat{n}}_{{\bf{k}}}^{{\bf{q}}}+{\hat{\bar{n}}}_{{\bf{k}}}^{{\bf{q}}})\end{array}),$$

where we have used ${{\bf{k}}}_{\pm }={\bf{k}}\pm {\bf{q}}\mathrm{/2}$, ${W}_{{\bf{k}}{\bf{q}}}^{\pm }={U}_{{{\bf{k}}}_{+}}{U}_{{{\bf{k}}}_{-}}\pm {V}_{{{\bf{k}}}_{+}}{V}_{{{\bf{k}}}_{-}}$ and ${w}_{{\bf{k}}{\bf{q}}}^{\pm }={U}_{{{\bf{k}}}_{+}}{V}_{{{\bf{k}}}_{-}}\pm {V}_{{{\bf{k}}}_{+}}{U}_{{{\bf{k}}}_{-}}$. Performing this change of basis on the equations of motion, we get:

22 $$\begin{array}{rcl}i\hslash \frac{d{\hat{y}}_{{\bf{k}}}^{{\bf{q}}}}{dt} & = & {\epsilon }_{{\bf{k}}{\bf{q}}}^{+}{\hat{s}}_{{\bf{k}}}^{{\bf{q}}}+\mathrm{(1}-{f}_{{{\bf{k}}}_{+}}-{f}_{{{\bf{k}}}_{-}})[{W}_{{\bf{k}}{\bf{q}}}^{-}(\delta {\hat{\Delta }}^{{\bf{q}}}+\delta {\hat{\bar{\Delta }}}^{{\bf{q}}}+{\phi }_{+}({\bf{q}}))\\  &  & -\,{w}_{{\bf{k}}{\bf{q}}}^{+}({g}_{0}[\delta {\hat{n}}_{\uparrow }^{{\bf{q}}}+\delta {\hat{n}}_{\downarrow }^{{\bf{q}}}]+{u}_{+}({\bf{q}}))]\end{array}$$

23 $$\begin{array}{rcl}i\hslash \frac{d{\hat{s}}_{{\bf{k}}}^{{\bf{q}}}}{dt} & = & {\epsilon }_{{\bf{k}}{\bf{q}}}^{+}{\hat{y}}_{{\bf{k}}}^{{\bf{q}}}+\mathrm{(1}-{f}_{{{\bf{k}}}_{+}}-{f}_{{{\bf{k}}}_{-}})[{W}_{{\bf{k}}{\bf{q}}}^{+}({\hat{\Delta }}^{{\bf{q}}}-{\hat{\bar{\Delta }}}^{{\bf{q}}}+{\phi }_{-}({\bf{q}}))\\  &  & -\,{w}_{{\bf{k}}{\bf{q}}}^{-}({g}_{0}[{\hat{n}}_{\uparrow }^{{\bf{q}}}-{\hat{n}}_{\downarrow }^{{\bf{q}}}]-{u}_{-}({\bf{q}}))]\end{array}$$

24 $$\begin{array}{rcl}i\hslash \frac{d{\hat{m}}_{{\bf{k}}}^{{\bf{q}}}}{dt} & = & -\,{\epsilon }_{{\bf{k}}{\bf{q}}}^{-}{\hat{h}}_{{\bf{k}}}^{{\bf{q}}}-({f}_{{{\bf{k}}}_{+}}-{f}_{{{\bf{k}}}_{-}})[{w}_{{\bf{k}}{\bf{q}}}^{-}({\hat{\Delta }}^{{\bf{q}}}-{\hat{\bar{\Delta }}}^{{\bf{q}}}+{\phi }_{-}({\bf{q}}))\\  &  & +\,{W}_{{\bf{k}}{\bf{q}}}^{+}({g}_{0}[{\hat{n}}_{\uparrow }^{{\bf{q}}}-{\hat{n}}_{\downarrow }^{{\bf{q}}}]-{u}_{-}({\bf{q}}))]\end{array}$$

25 $$\begin{array}{rcl}i\hslash \frac{d{\hat{h}}_{{\bf{k}}}^{{\bf{q}}}}{dt} & = & -{\epsilon }_{{\bf{k}}{\bf{q}}}^{-}{\hat{m}}_{{\bf{k}}}^{{\bf{q}}}+({f}_{{{\bf{k}}}_{+}}-{f}_{{{\bf{k}}}_{-}})[{w}_{{\bf{k}}{\bf{q}}}^{+}(\delta {\hat{\Delta }}^{{\bf{q}}}+\delta {\hat{\bar{\Delta }}}^{{\bf{q}}}+{\phi }_{+}({\bf{q}}))\\  &  & +\,{W}_{{\bf{k}}{\bf{q}}}^{-}({g}_{0}[\delta {\hat{n}}_{\uparrow }^{{\bf{q}}}+\delta {\hat{n}}_{\downarrow }^{{\bf{q}}}]+{u}_{+}({\bf{q}}))],\end{array}$$

where ${\phi }_{\pm }({\bf{q}})=\phi ({\bf{q}})\pm \bar{\phi }({\bf{q}})$, ${u}_{\pm }({\bf{q}})={u}_{\uparrow }({\bf{q}})\pm {u}_{\downarrow }({\bf{q}})$ and $\delta \hat{O}=\hat{O}-{\langle \hat{O}\rangle }_{T}$ (the subtraction of the mean-field average value matters only when *q* = 0). At the linear order, the sole effect of the drive fields is thus to shift the collective quantities which enter in the equations of motion:

26 $${\hat{\Delta }}^{{\bf{q}}}=\frac{{g}_{0}}{{L}^{3}}\,\sum _{{{\bf{k}}}_{1}}\,{\hat{d}}_{{{\bf{k}}}_{1}}^{{\bf{q}}}\to {\hat{\Delta }}^{{\bf{q}}}+\phi ({\bf{q}})$$

27 $${\hat{\bar{\Delta }}}^{{\bf{q}}}=\frac{{g}_{0}}{{L}^{3}}\,\sum _{{{\bf{k}}}_{1}}\,{\hat{\bar{d}}}_{{{\bf{k}}}_{1}}^{{\bf{q}}}\to {\hat{\bar{\Delta }}}^{{\bf{q}}}+\bar{\phi }({\bf{q}})$$

28 $${\hat{n}}_{\uparrow }^{{\bf{q}}}=\frac{1}{{L}^{3}}\,\sum _{{{\bf{k}}}_{1}}\,{\hat{n}}_{{{\bf{k}}}_{1}}^{{\bf{q}}}\to {\hat{n}}_{\uparrow }^{{\bf{q}}}+{u}_{\downarrow }({\bf{q}})/{g}_{0}$$

29 $${\hat{n}}_{\downarrow }^{{\bf{q}}}=\frac{1}{{L}^{3}}\,\sum _{{{\bf{k}}}_{1}}\,{\hat{\bar{n}}}_{{{\bf{k}}}_{1}}^{{\bf{q}}}\to {\hat{n}}_{\downarrow }^{{\bf{q}}}+{u}_{\uparrow }({\bf{q}})/{g}_{0}\mathrm{}.$$

Note that one recovers the zero temperature system Eqs. (14–16) of ref. ^38^ by setting ${f}_{{\bf{k}}}=0$ (in which case the equations of motion of the ${\hat{\gamma }}^{\dagger }\hat{\gamma }$ operators become trival).

## Linear Response to a Periodic Drive

### Matrix of response functions of a driven system

We now assume that the system is driven at a fixed frequency $\omega$, such that $\phi ({\bf{r}},t)=\phi ({\bf{r}}){e}^{i\omega t}$ and ${u}_{\sigma }({\bf{r}},t)={u}_{\sigma }({\bf{r}}){e}^{i\omega t}$. We can then replace the time derivatives $i\hslash {\partial }_{t}$ in Eqs. (22–25) by $\omega +i{0}^{+}$. Rederiving with respect to time and resumming the system to form the collective quantities (26–29) yields the 4-dimensional linear system

30 $$\left(\frac{V}{{g}_{0}}{\mathbb{I}}-\Pi (\omega ,{\bf{q}})\right)(\begin{array}{c}2i\Delta {\hat{\theta }}^{{\bf{q}}}\\ 2\delta |{\Delta }^{{\bf{q}}}|\\ {g}_{0}\delta {\hat{\rho }}^{{\bf{q}}}\\ {g}_{0}{\hat{p}}^{{\bf{q}}}\end{array})=\Pi (\omega ,{\bf{q}})(\begin{array}{c}{\phi }_{-}({\bf{q}})\\ {\phi }_{+}({\bf{q}})\\ {u}_{+}({\bf{q}})\\ -\,{u}_{-}({\bf{q}})\end{array}),$$

where ${\mathbb{I}}$ is the identity matrix. We have introduced the density and polarisation fluctuations and reparametrized the fluctuations of the order-parameter as:

31 $${\hat{\Delta }}^{{\bf{q}}}=(\Delta +\delta |{\Delta }^{{\bf{q}}}|){e}^{i{\hat{\theta }}^{{\bf{q}}}}$$

32 $${\hat{\bar{\Delta }}}^{{\bf{q}}}=(\Delta +\delta |{\Delta }^{{\bf{q}}}|){e}^{-i{\hat{\theta }}^{{\bf{q}}}}$$

33 $${\hat{\rho }}^{{\bf{q}}}={\hat{n}}_{\uparrow }^{{\bf{q}}}+{\hat{n}}_{\downarrow }^{{\bf{q}}}$$

34 $${\hat{p}}^{{\bf{q}}}={\hat{n}}_{\uparrow }^{{\bf{q}}}-{\hat{n}}_{\downarrow }^{{\bf{q}}}\mathrm{}.$$

We treat the phase ${\hat{\theta }}^{{\bf{q}}}$ of the order-parameter as an infinitesimal and therefore linearize the exponential in (31) and (32), which is consistent with our symmetry-breaking approach where the expansion is done around the mean-field state with a real Δ. The linear response matrix^1,42–44^, which relates the fluctuations of the density and order-parameter to the infinitesimal drive fields, is then

35 $$\chi =\frac{\Pi }{\frac{V}{{g}_{0}}{\mathbb{I}}-\Pi }\mathrm{}.$$

To describe the experimental behavior of a driven system, one usually concentrates on the imaginary part of $\chi$, which describes the energy absorbed by the system^1^ (whereas the real part describes the energy refracted or reflected by the system).

The matrix $\chi$ is expressed in terms of the 4 × 4 matrix $\Pi$ of the pair correlation functions^52^, computed in the BCS thermal state (4):

36 $$\begin{array}{rcl}\Pi  & = & (\begin{array}{cccc}{\Sigma }_{{W}^{+}{W}^{+}}^{\epsilon } & {\Sigma }_{{W}^{-}{W}^{+}}^{\omega } & -\,{\Sigma }_{{w}^{+}{W}^{+}}^{\omega } & 0\\ {\Sigma }_{{W}^{+}{W}^{-}}^{\omega } & {\Sigma }_{{W}^{-}{W}^{-}}^{\epsilon } & -\,{\Sigma }_{{w}^{+}{W}^{-}}^{\epsilon } & 0\\ -\,{\Sigma }_{{W}^{+}{w}^{+}}^{\omega } & -\,{\Sigma }_{{W}^{-}{w}^{+}}^{\epsilon } & {\Sigma }_{{w}^{+}{w}^{+}}^{\epsilon } & 0\\ 0 & 0 & 0 & -\,{\Sigma }_{{w}^{-}{w}^{-}}^{\epsilon }\end{array})\\  &  & -\,(\begin{array}{cccc}{S}_{{w}^{-}{w}^{-}}^{\epsilon } & {S}_{{w}^{+}{w}^{-}}^{\omega } & {S}_{{W}^{-}{w}^{-}}^{\omega } & 0\\ {S}_{{w}^{-}{w}^{+}}^{\omega } & {S}_{{w}^{+}{w}^{+}}^{\epsilon } & {S}_{{W}^{-}{w}^{+}}^{\epsilon } & 0\\ {S}_{{w}^{-}{W}^{-}}^{\omega } & {S}_{{w}^{+}{W}^{-}}^{\epsilon } & {S}_{{W}^{-}{W}^{-}}^{\epsilon } & 0\\ 0 & 0 & 0 & -{S}_{{W}^{+}{W}^{+}}^{\epsilon }\end{array})\end{array}$$

where we generalize the notations of refs. ^34,51^:

37 $${\Sigma }_{ab}^{\epsilon }(\omega ,{\bf{q}})=\sum _{{\bf{k}}}\,\frac{{\epsilon }_{{\bf{k}}{\bf{q}}}^{+}{a}_{{\bf{k}}{\bf{q}}}{b}_{{\bf{k}}{\bf{q}}}\mathrm{(1}-{f}_{{{\bf{k}}}_{+}}-{f}_{{{\bf{k}}}_{-}})}{{\omega }^{2}-{({\epsilon }_{{\bf{k}}{\bf{q}}}^{+})}^{2}}\,{\Sigma }_{ab}^{\omega }(\omega ,{\bf{q}})=\sum _{{\bf{k}}}\,\frac{\omega {a}_{{\bf{k}}{\bf{q}}}{b}_{{\bf{k}}{\bf{q}}}\mathrm{(1}-{f}_{{{\bf{k}}}_{+}}-{f}_{{{\bf{k}}}_{-}})}{{\omega }^{2}-{({\epsilon }_{{\bf{k}}{\bf{q}}}^{+})}^{2}}$$

38 $${S}_{ab}^{\epsilon }(\omega ,{\bf{q}})=\sum _{{\bf{k}}}\,\frac{{\epsilon }_{{\bf{k}}{\bf{q}}}^{-}{a}_{{\bf{k}}{\bf{q}}}{b}_{{\bf{k}}{\bf{q}}}({f}_{{{\bf{k}}}_{+}}-{f}_{{{\bf{k}}}_{-}})}{{\omega }^{2}-{({\epsilon }_{{\bf{k}}{\bf{q}}}^{-})}^{2}}\,{S}_{ab}^{\omega }(\omega ,{\bf{q}})=\sum _{{\bf{k}}}\,\frac{\omega {a}_{{\bf{k}}{\bf{q}}}{b}_{{\bf{k}}{\bf{q}}}({f}_{{{\bf{k}}}_{+}}-{f}_{{{\bf{k}}}_{-}})}{{\omega }^{2}-{({\epsilon }_{{\bf{k}}{\bf{q}}}^{-})}^{2}}\mathrm{}.$$

Here $a$ and $b$ are one of the functions ${W}^{+}$, ${W}^{-}$, ${w}^{+}$ or ${w}^{-}$ of ${\bf{k}}$ and ${\bf{q}}$, and ${\epsilon }_{{\bf{k}}{\bf{q}}}^{\pm }$ is short-hand for ${\epsilon }_{{\bf{k}}+{\bf{q}}\mathrm{/2}}\pm {\epsilon }_{{\bf{k}}-{\bf{q}}\mathrm{/2}}$. The first and second matrix in the right-hand side of (36) are the contribution of respectively the quasiparticle-quasiparticle and quasiparticle-quasihole continua to $\Pi$. In our unpolarized system, the polarization fluctuations ${\hat{n}}_{\uparrow }^{{\bf{q}}}-{\hat{n}}_{\downarrow }^{{\bf{q}}}$ are entirely decoupled from the other collective fields. Note that the response functions computed here for a driven system also give access, through a Laplace transform^34^, to the time response following a perturbation localized in time.

Remark that, up to some signs, the matrix $\Pi$ has a tensor-product structure when expressed in terms of the vector $({a}_{1},{a}_{2},{a}_{3},{a}_{4})=({W}^{+},{W}^{-},{w}^{+},{w}^{-})$:

39 $${\Pi }_{ij}=\{\begin{array}{ll}{\eta }_{ij}{\Sigma }_{{a}_{i},{a}_{j}}^{\epsilon }-{\eta {\prime} }_{ij}{S}_{{a}_{5-i},{a}_{5-j}}^{\epsilon } & {\rm{if}}\,i=j\,{\rm{or}}\,i=5-j\\ {\eta }_{ij}{\Sigma }_{{a}_{i},{a}_{j}}^{\omega }-{\eta {\prime} }_{ij}{S}_{{a}_{5-i},{a}_{5-j}}^{\omega } & {\rm{else}}\end{array}$$

The signs ${\eta }_{ij}=\pm \,1$ and ${\eta {\prime} }_{ij}=\pm \,1$ should be read on Eq. (36).

### Eigenenergies of the collective modes

The response of the system should diverge when the drive frequency coincides with the eigenfrequency ${\omega }_{{\bf{q}}}$ of a collective mode; to find those eigenfrequencies, one should thus search for the poles of $\chi$, in other words the zero of its denominator:

40 $${\rm{\det }}\left(\frac{V}{{g}_{0}}{\mathbb{I}}-\Pi ({z}_{{\bf{q}}},{\bf{q}})\right)=0.$$

When $\Pi$ has a branch cut on the real axis (which occurs for all $\omega \in {\mathbb{R}}$ at nonzero temperature and for $|\omega | > {{\rm{\min }}}_{{\bf{k}}}\,({\epsilon }_{{\bf{k}}+{\bf{q}}\mathrm{/2}}+{\epsilon }_{{\bf{k}}-{\bf{q}}\mathrm{/2}})$ at zero temperature), this equation cannot have a real solution. Its analytic continuation to the lower-half complex plane may however have solutions describing damped collective modes. Numerical and analytic methods to continue the matrix $\Pi$ through its branch cuts have been described in refs. ^20,21,34^. Note that the bare density-density response function ${\Pi }_{33}$ may have poles in the complex plane, which remain poles of the dressed response ${\chi }_{33}$.

### Explicit expressions of the response functions in the limit *g*_0_ → 0

In the limit of zero lattice spacing ($l\to 0$), ${g}_{0}$ tends to $0$, ${\Pi }_{11}$ and ${\Pi }_{22}$ are equivalent to $V/{g}_{0}$, while ${\Pi }_{33}$ and ${\Pi }_{44}$ have a finite limit (to interpret physically the elements of $\Pi$ the reader can use the correspondance $1,2,3,4\to \theta ,|\Delta |,\rho ,p$). We thus have the equivalences

41 $$\begin{array}{ccc}\Pi  & \mathop{\sim }\limits_{{g}_{0}\to 0} & (\begin{array}{cccc}V/{g}_{0} & {\Pi }_{12} & {\Pi }_{13} & 0\\ {\Pi }_{12} & V/{g}_{0} & {\Pi }_{23} & 0\\ {\Pi }_{13} & {\Pi }_{23} & {\Pi }_{33} & 0\\ 0 & 0 & 0 & {\Pi }_{44}\end{array}),\\ \Pi -\frac{V}{{g}_{0}}{\mathbb{I}} & \mathop{\sim }\limits_{{g}_{0}\to 0} & (\begin{array}{cccc}{\Pi }_{11}-V/{g}_{0} & {\Pi }_{12} & {\Pi }_{13} & 0\\ {\Pi }_{12} & {\Pi }_{22}-V/{g}_{0} & {\Pi }_{23} & 0\\ {\Pi }_{13} & {\Pi }_{23} & -\,V/{g}_{0} & 0\\ 0 & 0 & 0 & -\,V/{g}_{0}\end{array})\mathrm{}.\end{array}$$

Note that ${\Pi }_{11}-V/{g}_{0}$ and ${\Pi }_{22}-V/{g}_{0}$ have a finite limit when ${g}_{0}\to 0$. The determinant of the denominator of $\chi$ is then proportional to the determinant of the 2 × 2 upper left submatrix:

42 $$\begin{array}{lll}{\rm{\det }}\left(\Pi -\frac{V}{{g}_{0}}{\mathbb{I}}\right) & \mathop{\sim }\limits_{{g}_{0}\to 0} & {\left(\frac{V}{{g}_{0}}\right)}^{2}{\mathcal{D}}\,{\rm{with}}\\ {\mathcal{D}} & = & ({\Pi }_{11}-V/{g}_{0})({\Pi }_{22}-V/{g}_{0})-{({\Pi }_{12})}^{2}.\end{array}$$

Physically this means that the collective mode spectrum is entirely determined by the (modulus and phase) fluctuations of the order-parameter. However, the density responses may exhibit collective mode resonances as a result of the density-order parameter couplings. Using the equivalences (41) to compute the matrix product in $\chi$ we obtain:

43 $$\begin{array}{rcl}\tilde{\chi } & \equiv  & (\begin{array}{cccc}1 & 0 & 0 & 0\\ 0 & 1 & 0 & 0\\ 0 & 0 & V/{g}_{0} & 0\\ 0 & 0 & 0 & V/{g}_{0}\end{array})\,\chi \,(\begin{array}{cccc}{g}_{0}/V & 0 & 0 & 0\\ 0 & {g}_{0}/V & 0 & 0\\ 0 & 0 & 1 & 0\\ 0 & 0 & 0 & 1\end{array})\mathop{\sim }\limits_{{g}_{0}\to 0}-\frac{1}{{\mathcal{D}}}\\  &  & \times (\begin{array}{cc}{\tilde{\Pi }}_{22} & -\,{\Pi }_{12}\\ -\,{\Pi }_{12} & {\tilde{\Pi }}_{11}\\ ({\Pi }_{13}{\tilde{\Pi }}_{22}-{\Pi }_{12}{\Pi }_{23}) & ({\Pi }_{23}{\tilde{\Pi }}_{11}-{\Pi }_{13}{\Pi }_{12})\\ 0 & 0\end{array}\begin{array}{cc}{\Pi }_{13}{\tilde{\Pi }}_{22}-{\Pi }_{23}{\Pi }_{12} & 0\\ {\Pi }_{23}{\tilde{\Pi }}_{11}-{\Pi }_{13}{\Pi }_{12} & 0\\ ({\Pi }_{13}^{2}{\tilde{\Pi }}_{22}+{\Pi }_{23}^{2}{\tilde{\Pi }}_{11}-2{\Pi }_{13}{\Pi }_{23}{\Pi }_{12}-{\Pi }_{33}{\mathscr{D}}) & 0\\ 0 & -\,{\Pi }_{44}{\mathscr{D}}\end{array})\end{array}$$

We have used the notation $\tilde{\Pi }=\Pi -\frac{V}{{g}_{0}}$ and defined the response function matrix $\tilde{\chi }$ in the basis where all its coefficients are of order unity when ${g}_{0}\to 0$ (In this basis Eq. (30) reads $$(\begin{array}{cccc}2i\Delta {\hat{\theta }}^{{\bf{q}}} & 2\delta |{\Delta }^{{\bf{q}}}| & V\delta {\hat{\rho }}^{{\bf{q}}} & V{\hat{p}}^{{\bf{q}}}\end{array})\,=$$ $$\tilde{\chi }(\begin{array}{cccc}V{\phi }_{-}({\bf{q}})/{g}_{0} & V{\phi }_{+}({\bf{q}})/{g}_{0} & {u}_{+}({\bf{q}}) & -{u}_{-}({\bf{q}})\end{array})$$). For the density response functions we have in particular:

44 $${\tilde{\chi }}_{13}=\frac{{\Pi }_{23}{\Pi }_{12}-{\Pi }_{13}{\tilde{\Pi }}_{22}}{{\tilde{\Pi }}_{11}{\tilde{\Pi }}_{22}-{\Pi }_{12}^{2}}$$

45 $${\tilde{\chi }}_{23}=\frac{{\Pi }_{13}{\Pi }_{12}-{\Pi }_{23}{\tilde{\Pi }}_{11}}{{\tilde{\Pi }}_{11}{\tilde{\Pi }}_{22}-{\Pi }_{12}^{2}}$$

46 $${\tilde{\chi }}_{33}=\frac{2{\Pi }_{13}{\Pi }_{23}{\Pi }_{12}-{\Pi }_{13}^{2}{\tilde{\Pi }}_{22}-{\Pi }_{23}^{2}{\tilde{\Pi }}_{11}}{{\tilde{\Pi }}_{11}{\tilde{\Pi }}_{22}-{\Pi }_{12}^{2}}+{\Pi }_{33},$$

which coincides with the explicit expressions obtained by refs. ^21,45^ in the path-integral formalism. At weak coupling ($\Delta /\mu \to 0$) and $q=O(\Delta )$, the modulus-phase and modulus-density matrix elements ${\Pi }_{12}$ and ${\Pi }_{23}$ vanish such that the collective modes are either pure modulus modes (if their eigenenergy solves ${\Pi }_{22}({z}_{{\bf{q}}})-V/{g}_{0}=0$) or pure density-phase modes (if their eigenenergy solves ${\Pi }_{11}({z}_{{\bf{q}}})-V/{g}_{0}=0$).

### Angular points of the response functions

We conclude this section by remarking that the response functions have the same angular points as the spectral density

47 $$\rho (\omega )=\frac{\Pi (\omega +i{0}^{+})-\Pi (\omega -i{0}^{+})}{-\,2i\pi }\mathrm{}.$$

The quasiparticle-quasiparticle part of the spectral density (which originates from the first matrix in (36) with $\omega -({\epsilon }_{{\bf{k}}+{\bf{q}}\mathrm{/2}}+{\epsilon }_{{\bf{k}}-{\bf{q}}\mathrm{/2}})$ in the denominator) is nonzero when the “pair-breaking” resonance condition is satisfied

48 $$\omega ={\epsilon }_{{\bf{k}}+{\bf{q}}\mathrm{/2}}+{\epsilon }_{{\bf{k}}-{\bf{q}}\mathrm{/2}}\mathrm{}.$$

Physically, when this resonance condition is met, the drive field can break the pair of total momentum ${\bf{q}}$ into unpaired fermions of momenta ${\bf{k}}+{\bf{q}}\mathrm{/2}$ and ${\bf{k}}-{\bf{q}}\mathrm{/2}$. As a function of the increasing drive frequency $\omega$, this resonance condition is (*i*) satisfied by no wavevector when $\omega  < {\omega }_{1}$ (with ${\omega }_{1}=2\Delta$ at low-*q*), (*ii*) satisfied for a connected set of wavevectors around the dispersion minimum of the  BCS branch for ${\omega }_{1} < \omega  < {\omega }_{2}$, (*iii*) satisfied by two connected sets of wavevectors, one in the increasing and one in the decreasing part of the BCS branch for ${\omega }_{2} < \omega  < {\omega }_{3}$ and (*iv*) satisfied for a connected set of wavevectors in the increasing part of the branch for $\omega  > {\omega }_{3}$. These three boundary frequencies ${\omega }_{1}$, ${\omega }_{2}$ and ${\omega }_{3}$ will appear as angular points in the spectral function and therefore in the response functions.

The quasiparticle-quasihole part of the spectral density (which originates from the second matrix in (36) with $\omega -({\epsilon }_{{\bf{k}}+{\bf{q}}\mathrm{/2}}-{\epsilon }_{{\bf{k}}-{\bf{q}}/2})$ in the denominator) is nonzero when the “absorption-emission” resonance condition is satisfied

49 $$\omega ={\epsilon }_{{\bf{k}}+{\bf{q}}\mathrm{/2}}-{\epsilon }_{{\bf{k}}-{\bf{q}}\mathrm{/2}}\mathrm{}.$$

In this case, the drive field is not breaking the Cooper pairs but simply transferring more energy to the unpaired fermions already created by thermal agitation. This requires much less energy, which is why the quasiparticle-quasihole continuum is gapless. As a function of $\omega$, this resonance condition can be met on $(i)$ two disconnected sets of wavevectors, one in the increasing and one in the decreasing part of the BCS branch for $\omega  < {\omega }_{{\rm{ph}}}$, $(ii)$ a single connected set of wavevectors in the increasing part of the branch for $\omega  > {\omega }_{{\rm{ph}}}$. With ${\omega }_{{\rm{ph}}}$, we have found the four angular point of the response function $\omega \mapsto \chi (\omega )$ in $[0,+\,{\rm{\infty }}]$.

## Long Wavelength Limit

In the long wavelength limit ($q\ll \sqrt{2m\Delta }$) the solutions of the collective mode Eq. (40) and the behavior of the response functions can be studied analytically. Below the pair-breaking continuum (at energies lower than ${{\rm{\min }}}_{{\bf{k}}}\,({\epsilon }_{{\bf{k}}+{\bf{q}}\mathrm{/2}}+{\epsilon }_{{\bf{k}}-{\bf{q}}\mathrm{/2}})$ the problem has been studied in-depth at zero and nonzero temperature. At zero temperature a real solution of (40) corresponding to the Anderson-Bogoliubov sound branch can be found^37,46,47^. This branch appears as a pole in ${\rm{Re}}\,\chi$ and as a Dirac peak in ${\rm{Im}}\,\chi$ because the zero-temperature response functions are free of branch cuts below the pair-breaking continuum. This resonance was observed experimentally in the density-density response function by Bragg spectroscopy^13^ and in the low-*q* limit it can be identified with hydrodynamic first sound^8,12,22^. When the dispersion is supersonic (*i*.*e*. the function $q\mapsto {\omega }_{q}$ is convex) the resonance is broadened by intrinsic effects not captured by RPA^39,41^.

By contrast, the behavior of the response functions at high energy ($\omega  > {{\rm{\min }}}_{{\bf{k}}}({\epsilon }_{{\bf{k}}+{\bf{q}}\mathrm{/2}}+{\epsilon }_{{\bf{k}}-{\bf{q}}\mathrm{/2}})$) is known in literature only at zero temperature. There, the collective mode Eq. (40) has a complex root departing quadratically with $q$ from 2Δ^19^, and the modulus-modulus response shows correspondingly a resonance at energies above 2Δ^20^. Here, we show analytically that (within the RPA) the same resonance exists at nonzero temperature, even until the vicinity of ${T}_{c}$. This result is in agreement with Popov-Andrianov’s claim that the collective mode has the same energy at zero temperature and when $T\to {T}_{c}$.

### General case

We first compute the matrix elements ${\Pi }_{ij}$ on the upper-half complex plane, that is for ${\rm{Im}}\,z > 0$. The long wavelength expansion can be perform using the method exposed in ref. ^20^: since the energy is expected to depart quadratically from 2Δ, one parametrizes it as

50 $$z=2\Delta +\zeta \frac{\mu }{\Delta }\frac{{q}^{2}}{2m}.$$

The window $[2\Delta ,{\omega }_{2}]$ between the first two angular points of the branch cut corresponds in the limit $q\to 0$ to $\zeta \in [0,1]$. With respect to the zero temperature case^20^, the phase-phase and modulus-modulus matrix elements are simply multiplied by a factor $\tanh \,(\Delta /2T)$:

51 $${\check{\Pi }}_{11}(z,{\bf{q}})\mathop{\sim }\limits_{q\to 0}-\,\frac{{\pi }^{2}}{\check{q}}\,\tanh \left(\frac{\Delta }{2T}\right){f}_{11}(\zeta )\,{\rm{w}}{\rm{i}}{\rm{t}}{\rm{h}}\,{f}_{11}(\zeta )=\,{\rm{l}}{\rm{n}}\,\frac{1+\sqrt{1-\zeta }}{\sqrt{\zeta }}+\frac{i\pi }{2},$$

52 $$\begin{array}{ccc}{\check{\Pi }}_{22}(z,{\bf{q}}) & \mathop{\sim }\limits_{q\to 0} & {\pi }^{2}\check{q}\frac{\mu }{2\Delta }\,\tanh \left(\frac{\Delta }{2T}\right){f}_{22}(\zeta )\,{\rm{w}}{\rm{i}}{\rm{t}}{\rm{h}}\\ {f}_{22}(\zeta ) & = & \sqrt{1-\zeta }-\zeta \,{\rm{l}}{\rm{n}}\,\frac{1+\sqrt{1-\zeta }}{\sqrt{\zeta }}-\frac{i\pi \zeta }{2},\end{array}$$

where we have replaced the sums over **k** in (37) and (38) by an integral $\int \,{d}^{3}k/{(2\pi /L)}^{3}$ and we have introduced the dimensionless quantities $\check{q}=q/\sqrt{2m\Delta }$ and ${\check{\Pi }}_{ij}=\alpha ({\Pi }_{ij}-{L}^{3}/{g}_{0})$ for $(i,j)=(1,1)$ or $(2,2)$ and ${\check{\Pi }}_{ij}=\alpha {\Pi }_{ij}$ for the other matrix elements, the nondimensionalization factor $\alpha$ being $\Delta {\mathrm{(2}\pi /L\sqrt{2m\Delta })}^{3}$ (the dimensionless response functions ${\check{\chi }}_{ij}$ will accordingly be multiplied by the appropriate power of *α*: *α*^−1^ for ${\chi }_{11}$, ${\chi }_{22}$ and ${\chi }_{12}$, *α*^0^ for ${\chi }_{13}$ and ${\chi }_{23}$ and *α*^1^ for ${\chi }_{33}$). We have written the complex functions ${f}_{11}$ and ${f}_{22}$ of $\zeta$ in such a way that their spectral density (their imaginary part in the limit ${\rm{Im}}\,\zeta \to {0}^{+}$) is directly given by their last term. The modulus-phase matrix element is independent of $\zeta$ (it can be approximated by its value in $q=0$, $z=2\Delta$) but, unlike ${\Pi }_{11}$ and ${\Pi }_{22}$, depends on temperature through the two ratios $\Delta /T$ and $\mu /T$:

53 $${\check{\Pi }}_{12}(z,{\bf{q}})\mathop{\sim }\limits_{q\to 0}{\check{\Pi }}_{12}(2\Delta ,0)={\pi }^{2}\sqrt{\frac{\mu }{2\Delta }}\,\tanh \,\left(\frac{\Delta }{2T}\right){g}_{12}\left(\frac{\mu }{T},\frac{\Delta }{T}\right),$$

where we have introduced the improper integral (${\mathcal{P}}$ denotes the Cauchy principal part):

54 $${\pi }^{2}\sqrt{\frac{M}{2D}}\,\tanh \,\left(\frac{D}{2}\right){g}_{12}(M,D)=-\,\pi {\mathcal{P}}\,{\int }_{-M/D}^{\infty }\,\frac{d\xi \sqrt{\xi +M/D}}{\xi \sqrt{{\xi }^{2}+1}}\,\tanh \,\frac{D\sqrt{{\xi }^{2}+1}}{2}\mathrm{}.$$

Note that the quasiparticle-quasihole integrals (38) have been negligible in deriving expressions (51–53).

To find a root of the collective mode Eq. (40), one analytically continues ${\Pi }_{11}$ and ${\Pi }_{22}$ (and hence the determinant of $\Pi$) from upper to lower half-complex plane (the forms given in Eqs. (51) and (52) are in fact already analytic for ${\rm{Re}}\,\zeta \in [0,1]$). Eq. (40) for the complex collective mode frequency ${z}_{q}=2\Delta +{\zeta }_{s}\frac{\mu }{\Delta }\frac{{q}^{2}}{2m}+O({q}^{3})$ becomes an explicit (yet transcendental) equation for the reduced frequency ${\zeta }_{s}$

55 $${f}_{11}({\zeta }_{s}){f}_{22}({\zeta }_{s})+{g}_{12}^{2}\left(\frac{\mu }{T},\frac{\Delta }{T}\right)=0.$$

At zero temperature, this equation was derived in ref. ^19^ in the weak-coupling limit and ref. ^20^ in the general case. In this low-*q* limit, the only dependence on temperature is through the $\zeta$-independent second term of (55).

### Close to the phase transition

Unlike in the phononic regime ($q\to 0$ with $z=cq$)^21^, no dramatic phenomenon occurs for the collective mode of the pair-breaking continuum when $T$ tends to the critical temperature ${T}_{c}$. We recall that in the RPA, the limit of the phase transition from the superfluid phase corresponds to

56 $$\frac{\Delta }{T}\to 0$$

57 $$\frac{\mu }{T}=\frac{\mu ({T}_{c})}{{T}_{c}}+O{\left(\frac{\Delta }{T}\right)}^{2}.$$

The RPA also assumes an infinite fermionic quasiparticle lifetime and thus describes the collective modes and their damping by the fermionic continua in the collisionless approximation.

The function ${g}_{12}$ tends to a finite nonzero constant in the limit $T\to {T}_{c}$:

58 $${g}_{12}\left(\frac{\mu }{T},\frac{\Delta }{T}\right)\mathop{\to }\limits_{T\to {T}_{c}}{g}_{\mathrm{12,}c}\left(\frac{{\mu }_{c}}{{T}_{c}}\right)\equiv -\,\frac{2}{\pi }\sqrt{\frac{2{T}_{c}}{{\mu }_{c}}}{\mathcal{P}}\,{\int }_{-{\mu }_{c}/{T}_{c}}^{+\infty }\,\frac{\tanh \,\frac{|e|}{2}\sqrt{e+\frac{{\mu }_{c}}{{T}_{c}}}de}{e|e|},$$

where we denote ${\mu }_{c}\equiv \mu ({T}_{c})$. In fact, the resonance near ${T}_{c}$ for a given value of $\mathrm{1/}{k}_{{\rm{F}}}a$ has exactly the same shape as the $T=0$ resonance for a lower value (corresponding to weaker-coupling) of $\mathrm{1/}{k}_{{\rm{F}}}a$. Using an equation-of-state to relate $\mathrm{1/}{k}_{{\rm{F}}}a$ to both ${\mu }_{c}/{T}_{c}$ and $\mu (T=0)/\Delta (T=0)$^21^, the corresponding values ${a}_{0}$ and ${a}_{c}$ of the scattering length at $T=0$ and ${T}_{c}$ are found by solving:

59 $${g}_{\mathrm{12,}c}\left({\frac{{\mu }_{c}}{{T}_{c}}|}_{a={a}_{c}}\right)=\mathop{\mathrm{lim}}\limits_{T\to 0}\,{g}_{12}\left({\frac{\mu }{\Delta }|}_{a={a}_{0}}\frac{\Delta }{T},\frac{\Delta }{T}\right)\mathrm{}.$$

Finally, the explicit expressions of the response functions in the long wavelength limit, at arbitrary $0\le T < {T}_{c}$, and in the limit $T\to {T}_{c}$ are:

60 $$\begin{array}{ccc}{\check{\chi }}_{11}(z,{\bf{q}}) & = & -\frac{\check{q}}{{\pi }^{2}\,\tanh \,\frac{\Delta }{2T}}\frac{{f}_{22}(\zeta )}{{f}_{11}(\zeta ){f}_{22}(\zeta )+{g}_{12}^{2}(\mu /T,\Delta /T)}+O({q}^{2})\mathop{\sim }\limits_{T\to {T}_{c}}\\  &  & -\,\frac{2\check{q}}{{\pi }^{2}}\frac{{T}_{c}}{\Delta }\frac{{f}_{22}(\zeta )}{{f}_{11}(\zeta ){f}_{22}(\zeta )+{g}_{12,c}^{2}({\mu }_{c}/{T}_{c})}\end{array}$$

61 $$\begin{array}{ccc}{\check{\chi }}_{22}(z,{\bf{q}}) & = & \frac{1}{\check{q}{\pi }^{2}\,\tanh \,\frac{\Delta }{2T}}\frac{2\Delta }{\mu }\frac{{f}_{11}(\zeta )}{{f}_{11}(\zeta ){f}_{22}(\zeta )+{g}_{12}^{2}(\mu /T,\Delta /T)}+O(1)\mathop{\sim }\limits_{T\to {T}_{c}}\\  &  & \frac{4}{{\pi }^{2}\check{q}}\frac{{T}_{c}}{{\mu }_{c}}\frac{{f}_{11}(\zeta )}{{f}_{11}(\zeta ){f}_{22}(\zeta )+{g}_{12,c}^{2}({\mu }_{c}/{T}_{c})}\end{array}$$

62 $$\begin{array}{ccc}{\check{\chi }}_{12}(z,{\bf{q}}) & = & -\,\frac{1}{{\pi }^{2}\,\tanh \,\frac{\Delta }{2T}}\sqrt{\frac{2\Delta }{\mu }}\frac{{g}_{12}(\mu /T,\Delta /T)}{{f}_{11}(\zeta ){f}_{22}(\zeta )+{g}_{12}^{2}(\mu /T,\Delta /T)}+O(q)\mathop{\sim }\limits_{T\to {T}_{c}}\\  &  & -\,\frac{2\sqrt{2}}{{\pi }^{2}}\frac{{T}_{c}}{\sqrt{{\mu }_{c}\Delta }}\frac{{g}_{\mathrm{12,}c}({\mu }_{c}/{T}_{c})}{{f}_{11}(\zeta ){f}_{22}(\zeta )+{g}_{\mathrm{12,}c}^{2}({\mu }_{c}/{T}_{c})}\mathrm{}.\end{array}$$

Thus, the response functions have exactly the same shape (they coincide up to a proportionality factor) near ${T}_{c}$ as they have at $T=0$ for the slightly different value of the interaction strength given by (59).

**Figure 1:**
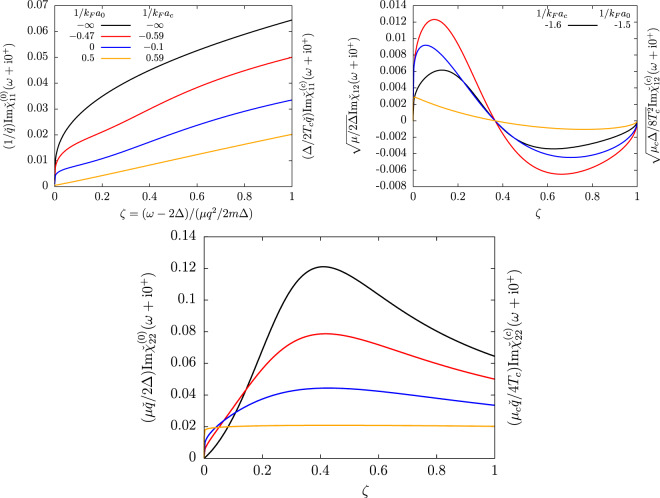
The order-parameter response functions (top left pannel: phase-phase, top right pannel: modulus-phase, bottom pannel: modulus-modulus response) are shown as functions of the reduced drive frequency $\zeta =(\omega -2\Delta )/(\mu {q}^{2}/2m\Delta )$ of Eq. (50) in the long wavelength limit after multiplication by the power of $q$ which ensures a finite non zero limit when $q\to 0$. Their value at zero temperature (${\chi }_{ij}^{\mathrm{(0)}}$) coincide up to a proportionality factor (shown in the $y$-axis labels) with their value near the phase transition (${\chi }_{ij}^{(c)}$) at a slightly different interaction strength $\mathrm{1/}{k}_{F}a$ obtain using the correspondence Eq. (59). The values of $\mathrm{1/}{k}_{F}a$ used to span the BCS side of the crossover at $T=0$ correspond with the mean-field equation-of-state to $\mu /\Delta =+\,\infty$, $2$, $0.86$ and $0.07$ (respectively black, blue, red and orange lines). At $T\to {T}_{c}$ they correspond to ${\mu }_{c}/{T}_{c}=+\,\infty$, $4.2$, $1.8$ and $0.15$. For ${\chi }_{12}$, the BCS limit $\mu /\Delta ,{\mu }_{c}/{T}_{c}\to +\,\infty$ is reached logarithmically such that we have used the finite values $\mu /\Delta =10$, $1/{k}_{F}{a}_{0}=-\,1.5$ at $T=0$ and ${\mu }_{c}/{T}_{c}=20.6$, $1/{k}_{F}{a}_{c}=-\,1.6$ near ${T}_{c}$. Note that the response functions are rescaled by the power of *q* ensuring a finite non zero limit when $q\to 0$. Similarly the response functions near ${T}_{c}$ are rescaled by the right power of $\Delta /{T}_{c}$. Finally, a proportionality factor (depending of $\mu /\Delta$ at $T=0$ and ${\mu }_{c}/{T}_{c}$ near ${T}_{c}$) is applied to ensure that the response functions at $T=0$ and $T\to {T}_{c}$ fall on the same line.

In Fig. 1, we show how the shape of the order-parameter response functions (${\chi }_{11}$, ${\chi }_{22}$ and ${\chi }_{12}$) change when going from the BCS limit ($1/{k}_{F}a\to -\,\infty$ that is $\mu /\Delta \to +\,\infty$ at $T=0$ or ${\mu }_{c}/{T}_{c}\to +\,\infty$ at $T={T}_{c}$) to the threshold of the BEC regime where $\mu$ vanishes. Exploiting the equivalence (59), the figure describes together the crossover at $T=0$ and $T\to {T}_{c}$. Irrespectively of the interaction regime, the phase-phase response is a monotonously increasing function of the drive frequency and only reflects the incoherent response of the pair-breaking continuum, without collective effects. Conversely, both the modulus-modulus and modulus-phase response functions display a maximum that can be interpreted as a collective mode in the BCS limit (black curves) and up until unitarity (blue curves). As explained in ref. ^20^, this maximum can be fitted to extract the frequency and damping rate of the collective mode to a good precision. The fit function to use is $\omega \mapsto \,{\rm{Im}}\,({Z}_{q}/(\omega -{z}_{q})+{C}_{q})$, where the complex parameters ${z}_{q}$, ${Z}_{q}$ and ${C}_{q}$ represent respectively the complex energy of the collective mode, its residue, and an incoherent flat background. A remarkable effect of this background ${C}_{q}$ is to displace the location of the maximum of ${\chi }_{22}$ and ${\chi }_{12}$ to respectively $\zeta \simeq 0.4$ and $\zeta \simeq 0.1$ in a very broad interaction range. The variations of ${\zeta }_{s}$ (which decreases when increasing the coupling strength) are thus not visible by simply looking at the maximum location. Soon after unitarity, the resonance in ${\chi }_{22}$ and ${\chi }_{12}$ disappears and only a sharp feature near $\omega =2\Delta$ remains. This abrupt lower edge of the continuum is in $\zeta =0$ so it is not departing quadratically with $q$ from 2Δ (see also the color Fig. 6 in the BEC regime) as the Popov-Andrianov resonance does in the BCS regime, and it can no longer be interpreted as a collective mode. As understood in ref. ^20^, this is because the complex root ${z}_{q}$ of the collective mode Eq. (40) has a real part below $2\Delta$ (i.e. ${\rm{Re}}\,{\zeta }_{s} < 0$) and does no longer trigger a resonance inside the pair-breaking continuum.

### Density matrix elements in the long wavelength limit

We now study the density responses of the system ${\chi }_{i3}$, $i=1,2,3$ in the long wavelength limit $q\to 0$ at energies above but close to 2Δ. For $q\to 0$ at fixed $\zeta$, the three matrix elements needed to compute the density response functions are given by:

63 $$\delta {\check{\Pi }}_{13}\equiv {\check{\Pi }}_{13}+{\check{\Pi }}_{11}={\pi }^{2}\frac{\mu }{2\Delta }\,\tanh \,\left(\frac{\Delta }{2T}\right){f}_{13}(\zeta )\check{q}+O({q}^{2})\,{\rm{w}}{\rm{i}}{\rm{t}}{\rm{h}}\,{f}_{13}(\zeta )=\sqrt{1-\zeta }$$

64 $$\delta {\check{\Pi }}_{23}\equiv {\check{\Pi }}_{23}+{\check{\Pi }}_{12}={\pi }^{2}{\left(\frac{\mu }{2\Delta }\right)}^{3/2}\,\tanh \,\left(\frac{\Delta }{2T}\right)\left[\zeta ,{g}_{12},(\frac{\mu }{T},\frac{\Delta }{T}),+,{g}_{23},(\frac{\mu }{T},\frac{\Delta }{T})\right]{\check{q}}^{2}+O({q}^{3})$$

65 $$\delta {\check{\Pi }}_{33}\equiv {\check{\Pi }}_{33}+{\check{\Pi }}_{11}+2{\check{\Pi }}_{13}={g}_{33}\left(\frac{\mu }{T},,,\frac{\Delta }{T}\right){\check{q}}^{2}+\frac{{\pi }^{2}}{2}\frac{{\mu }^{2}}{4{\Delta }^{2}}\,\tanh \,\left(\frac{\Delta }{2T}\right){f}_{33}(\zeta ){\check{q}}^{3}+O({q}^{4})$$

66 $${\rm{with}}\,{f}_{33}=(\zeta -2)\sqrt{1-\zeta }-{\zeta }^{2}\,\mathrm{ln}\,\frac{1+\sqrt{1-\zeta }}{\sqrt{\zeta }}-\frac{i\pi {\zeta }^{2}}{2}$$

67 $$\begin{array}{ccc}{\pi }^{2}{\left(\frac{M}{2D}\right)}^{3/2}\,\tanh \,\left(\frac{D}{2}\right){g}_{23}(M,D) & = & \frac{\pi }{3}{\mathcal{P}}\,{\int }_{-M/D}^{{\rm{\infty }}}\,\frac{d\xi {\sqrt{\xi +M/D}}^{3}}{\xi {\epsilon }^{3}}\,\tanh \,\frac{D\epsilon }{2}\\  &  & +\,\frac{\pi D}{6}{\mathcal{P}}\,{\int }_{-M/D}^{{\rm{\infty }}}\,\frac{\xi d\xi {\sqrt{\xi +M/D}}^{3}}{{\epsilon }^{2}\,{\cosh }^{2}\,\frac{D\epsilon }{2}}\end{array}$$

68 $$\begin{array}{ccc}{\rm{a}}{\rm{n}}{\rm{d}}\,{g}_{33}(M,D) & = & \frac{\pi }{16}{\mathcal{P}}\,{\int }_{-M/D}^{{\rm{\infty }}}\,\frac{d\xi \sqrt{\xi +M/D}}{{\cosh }^{2}\frac{D\epsilon }{2}{\epsilon }^{5}}\\  &  & \times [-\,2D\epsilon \left(\xi ,{\epsilon }^{2},+,\frac{2}{3},[,1,-,2,{\xi }^{2},(,1,+,{\epsilon }^{2},),],(,\xi ,+,M,/,D,)\right)\\  &  & +\,\frac{\sinh \,\frac{3D\epsilon }{2}}{\cosh \,\frac{D\epsilon }{2}}(\xi {\epsilon }^{2}+2(\xi +M/D))\\  &  & +\,\tanh \,\frac{D\epsilon }{2}\left(\xi ,{\epsilon }^{2},+,2,(,\xi ,+,M,/,D,),(1+\frac{2}{3}{D}^{2}{\xi }^{2}{\epsilon }^{2})\right)].\end{array}$$

Those expressions, like those of the modulus and phase matrix elements (51–53) are obtained by treating separately the resonant wavevectors (for the resonance condition $z={\epsilon }_{{\bf{k}}+{\bf{q}}\mathrm{/2}}+{\epsilon }_{{\bf{k}}-{\bf{q}}\mathrm{/2}}$), located in this limit around the minimum ${k}_{0}$ of the BCS branch. For those wavevectors, we set

69 $$k={k}_{0}+Kq,$$

and expand the integrand in (37) at fixed *K*. This yields the leading order contribution to ${\Pi }_{11}$, ${\Pi }_{22}$ and $\delta {\Pi }_{13}$. For ${\Pi }_{12}$, $\delta {\Pi }_{23}$ and $\delta {\Pi }_{33}$ the leading order is dominated by the wavevectors away from ${k}_{0}$ and is obtained by expanding directly in powers of $q$ at fixed $k$ (with a contribution of the quasiparticle-quasihole integrals from Eq. (38)). For $\delta {\Pi }_{33}$ specifically, the subleading order $O({q}^{3})$ (which matters for the imaginary part of the response function ${\rm{Im}}\,{\chi }_{33}$), is obtained by subtracting the leading one and then using the reparametrisation of the wavevectors, Eq. (69).

Using the expansions Eqs. (63–65), we obtain the expressions of the density response functions:

70 $${\check{\chi }}_{13}=1+\frac{\mu }{2\Delta }\frac{(\zeta {g}_{12}+{g}_{23}){g}_{12}-{f}_{13}{f}_{22}}{{f}_{11}{f}_{22}+{g}_{12}^{2}}{\check{q}}^{2}+O({q}^{3})$$

71 $${\check{\chi }}_{23}=-\sqrt{\frac{\mu }{2\Delta }}\frac{{f}_{13}{g}_{12}+(\zeta {g}_{12}+{g}_{23}){f}_{11}}{{f}_{11}{f}_{22}+{g}_{12}^{2}}\check{q}+O({q}^{2})$$

72 $$\begin{array}{ccc}{\check{\chi }}_{33} & = & {g}_{33}{\check{q}}^{2}+{\pi }^{2}\frac{{\mu }^{2}}{4{\Delta }^{2}}\,\tanh \,\left(\frac{\Delta }{2T}\right)\\  &  & \times \,\left[\frac{{f}_{33}}{2}\,-\,\frac{2{f}_{13}(\zeta {g}_{12}+{g}_{23}){g}_{12}-{f}_{13}^{2}{f}_{22}+{(\zeta {g}_{12}+{g}_{23})}^{2}{f}_{11}}{{f}_{11}{f}_{22}+{g}_{12}^{2}}\right]{\check{q}}^{3}+O({q}^{4}),\end{array}$$

where we omit the evaluation of the functions ${f}_{ij}$ and ${g}_{ij}$ respectively in $\zeta$ and $(\mu /T,\Delta /T)$. The limiting behaviour near the phase transition follows immediately by using the limiting behaviour of Δ and $\mu$ from Eqs. (56–57) and replacing ${g}_{12}(\mu /T,\Delta /T)$ and ${g}_{23}(\mu /T,\Delta /T)$ by their finite nonzero limit ${g}_{\mathrm{12,}c}({\mu }_{c}/{T}_{c})$ and ${g}_{\mathrm{23,}c}({\mu }_{c}/{T}_{c})$ with

73 $${g}_{23,c}(M)=\frac{2\sqrt{2}}{3\pi {M}^{3/2}}\,{\int }_{-M}^{+\infty }\,\frac{de{(e+M)}^{3/2}}{e\,{\cosh }^{2}(e/2)}.$$

The function ${g}_{33}$, which gives only a $\zeta$-independent shift of ${\rm{Re}}\,{\chi }_{33}$, diverges asymptotically as $O{(\Delta /{T}_{c})}^{-\mathrm{3/2}}$ near the phase transition.

**Figure 2:**
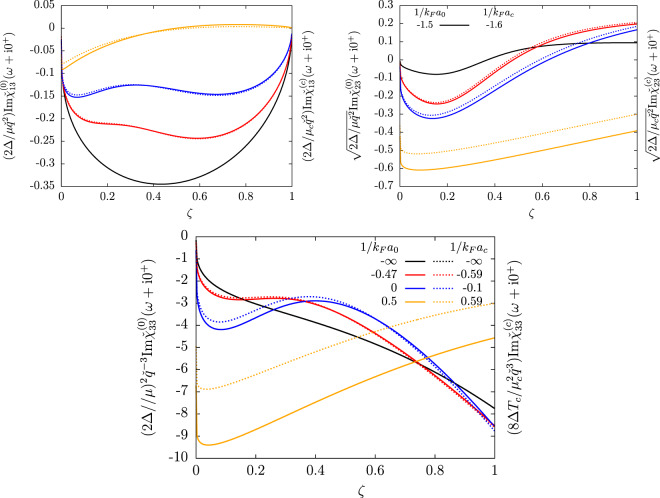
The density response functions (top left pannel: density-phase, top right pannel: density-modulus, bottom pannel: density-density response) are shown as functions of the reduced drive frequency $\zeta =(\omega -2\Delta )/(\mu {q}^{2}/2m\Delta )$ of Eq. (50) in the long wavelength limit after multiplication by the power of $q$ which ensures a finite non zero limit when $q\to 0$. Their values at zero temperature (${\chi }_{ij}^{\mathrm{(0)}}$, solid lines) are compared to their value near the phase transition (${\chi }_{ij}^{(c)}$, dashed lines) after the appropriate rescaling and the change of interaction strength which brings the order-parameter responses on the same line (see Fig. 1, and the correspondence Eq. (59)). Please refer to the caption of Fig. 1 for the values of $\mu /\Delta$ and of ${\mu }_{c}/{T}_{c}$ corresponding to the chosen values of $1/{k}_{F}a$.

In Fig. 2, we show the density response functions on the BCS side of the crossover. Unlike for the order-parameter response functions, no exact correspondance between zero temperature and the transition temperature can be found by changing the interaction strength (this is due to the temperature dependence of ${g}_{23}$), so we show separately the functions at $T=0$ (in solid curves) and at $T\to {T}_{c}$ (in dashed curves). The difference between the $T=0$ and $T\to {T}_{c}$ curves (after the appropriate rescaling) remains however fairly small, and tends to 0 in the BCS limit (black curves). Remarkably, a minimum characteristic of the Popov-Andrianov collective mode is visible in all three density responses. In ${\chi }_{23}$, this minimum is a global minimum (for $\zeta \in [0,1]$) which exist (as in ${\chi }_{22}$ and ${\chi }_{12}$) from the BCS limit up until unitarity. For the density-density and density-phase responses ${\chi }_{33}$ and ${\chi }_{13}$, this minimum is a local minimum, which exists close to unitarity (blue curves) around $\zeta =0.1$. Because of the decoupling between the phase-density fluctuations and the modulus fluctuations in the weak-coupling limit, this minimum disappears from ${\chi }_{13}$ and ${\chi }_{33}$ when $\mathrm{1/}{k}_{F}a\to -\,\infty$ (black curves). After unitary, when approaching the BEC regime (orange curves), the resonances in all three density responses are replaced by a sharp edge in $\omega \to 2{\Delta }^{+}$ ($\zeta \to {0}^{+}$). This is the same phenomenon as in the order-parameter response functions.

### Coexistence with the phononic collective modes near *T*_*c*_

**Figure 3:**
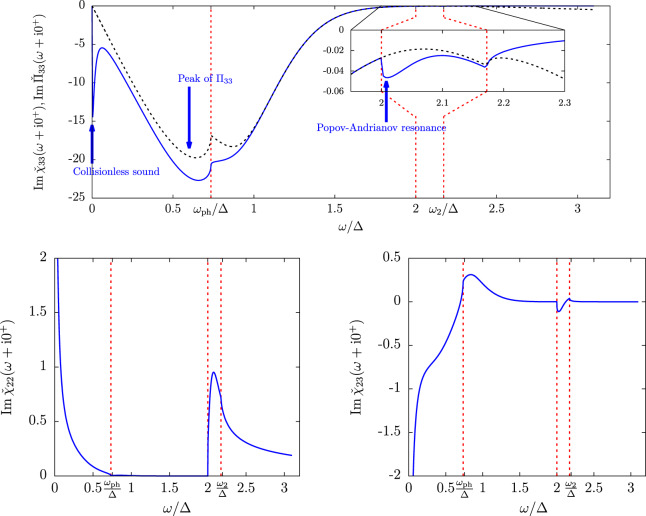
The density-density (top pannel), modulus-modulus (bottom left pannel) and modulus-density (bottom right pannel) response functions shown in function of the drive frequency $\omega$. The curves were drawn in the strong coupling regime ($\mathrm{1/}{k}_{{\rm{F}}}a\simeq -\,0.1$, ${\mu }_{c}/{T}_{c}\simeq 1.8$), near the transition temperature ($\Delta /T=0.1$) and at long wavelength ($\check{q}=0.1$). The dimensionless response functions are shown this time without rescaling and from $\omega =0$ until far inside the pair-breaking continuum. The vertical dashed lines show the angular points of the fermionic continua, from left to right ${\omega }_{{\rm{ph}}}$, ${\omega }_{1}$(=$2\Delta$) and ${\omega }_{2}$. On the top panel, the black dashed line is the pure density contribution ${\rm{Im}}\,{\Pi }_{33}$ to the density-density response (see Eq. (46)) and the inset is a zoom on the behaviour near $\omega =2\Delta$.

To compare the Popov-Andrianov resonance to the other collective effects of a superfluid Fermi gases near ${T}_{c}$, we show in Fig. 3 the response functions from $\omega =0$ up until $\omega  > 3\Delta$ in the strong coupling regime and temperature close to ${T}_{c}$. The sharpest feature in both the order-parameter and density responses is the resonance, at very low energy (that is at $\omega =uq$ with a velocity $u\propto \sqrt{{T}_{c}-T}$), of the collisionless phononic collective mode found in ref. ^21^. Still at phononic energies $\omega \propto q$, the density-density response function shows a broad peak caused entirely by ${\Pi }_{33}$ (shown as a black dashed line) and also noticed in ref. ^21^. This is simply the peak of the Lindhard function, which exists also in the normal phase. Finally, inside the first window $[2\Delta ,{\omega }_{2}]$ of analyticity of the pair-breaking continuum, all response functions show the peak characteristic of the Popov-Andrianov resonance, whose shape matches the one shown on Figs. 1 and 2. Due to the absence of rescaling with the wavevector *q* in Fig. 3, the peak is much more intense in the modulus-modulus response, and to a lesser extent in the modulus-density response, than in the density-density response.

### Experimental protocol

Our results suggest a very simple experimental protocol to observe the resonance: using a Bragg spectroscopic measurement as in ref. ^13^, one should observe that the first extremum above 2Δ varies quadratically (both in location and width) with $q$, a behavior which can be viewed as the fingerprint of the Popov-Andrianov-Higgs mode. The optimal interaction regime is around unitarity and the optimal wavevector is around $0.5\times \sqrt{2m\Delta }$ ($q$ should not be too small to avoid the ${q}^{3}$ cancellation of ${\chi }_{33}$ near 2Δ but not too large either otherwise the minimum is reabsorbed by the continuum edge, see the lower panels of Fig. 5).

Alternatively, the resonance could be observed through the modulus-density response function ${\chi }_{23}$ by (*i*) exciting the order-parameter modulus $\delta |\Delta |$ through a modulation of the scattering length at frequency $\omega$ and wavelength $2\pi /q$ and (*ii*) measuring the intensity of the density modulation $\delta \rho$ at wavelength $2\pi /q$. This should be easier than the scheme of ref. ^20^ which proposed to measure ${\chi }_{22}$ by interferometry. Using the symmetry of the response matrix $\chi$, one can also excite the density $\delta \rho$ (by a Bragg pulse^13^ or using the trapping potential^22^) and measure the order-parameter modulus $\delta |\Delta |$ either by interferometry or by bosonizing the Cooper pairs through a fast sweep of the scattering length, as was done in ref. ^23^.

## At Shorter Wavelengths

Outside the long wavelength limit, that is $q\approx \sqrt{2m\Delta },\sqrt{2m\mu }$ when $\mu$ and Δ are comparable (see Eq. (90) in ref. ^34^ for a more detailed discussion of the limit of validity of the long-wavelength limit), we study the response functions by performing numerically the integral over internal wavevectors **k** in Eqs. (37) and (38) (see Appendix B for more details on the numerical implementation).

### At zero temperature

#### Weak-coupling regime

**Figure 4:**
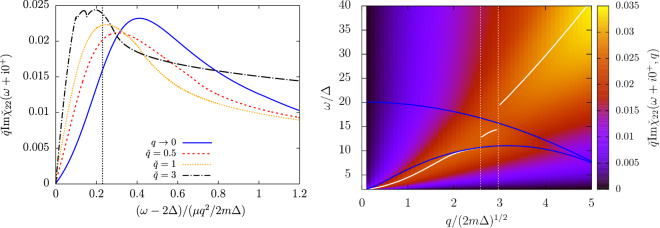
Left panel: the modulus-modulus response function is shown as a function of $(\omega -2\Delta )/(\mu {q}^{2}/2m\Delta )$ at weak-coupling $\mu /\Delta =10$. The wavevector $q$ varies from $q\to 0$ (solid blue line, see section V), $\check{q}=0.5$ (red dashed line), $\check{q}=1$ (orange dotted line) to $\check{q}=3$ (black dash-dotted line), and the response function is multiplied by $\check{q}$. The vertical dotted line indicates the reduced eigenergy ${\rm{Re}}\,{\zeta }_{s}\simeq 0.23$ of the pole found (when $q\to 0$) in the analytic continuation (see Eq. 55) through $[2\Delta ,{\omega }_{2}]$. Right panel: the same evolution is shown in colors as functions of both the wavevector $q$ on the $x$-axis and drive frequency $\omega$ on the $y$-axis. The response function is still multiplied by $\check{q}$. Superimposed to the color plot, the angular points ${\omega }_{2}$ (lower blue solid line) and ${\omega }_{3}$ (upper blue solid line), and the location of the global maximum of the function $\omega \mapsto \mathrm{Im}{\chi }_{22}$ (white solid line). As $q$ increases, this maximum jumps from the interval $[2\Delta ,{\omega }_{2}]$ where it is located at low $q$, to $[{\omega }_{2},{\omega }_{3}]$ and eventually to $[{\omega }_{3},+\,\infty [$ at large $q$. Each jump is marked by a vertical white dotted line.

On the left panel of Fig. 4, we show the modulus-modulus response at relatively weak-coupling ($\mu /\Delta =10$) and zero temperature as a function of $\omega$ (rescaled as in the long wavelength section) for increasing values of the wavevector $q$. On the right panel, we show the same dispersion relation but in colors, with $q$ on the $x$-axis and $\omega$ on the $y$-axis. The Popov-Andrianov resonance we have characterized at low $q$ remains as a broader and shallower maximum as $q$ increases (see the rescaling of the $x$ and $y$-axis on the left panel of Fig. 4) that travels roughly quadratically through the continuum. In the modulus-modulus response function, the augmentation of wavevector is thus unfavourable for the observation of the resonance in the pair-breaking continuum. Note that the location of the maximum is discontinuous when crossing ${\omega }_{2}$ and ${\omega }_{3}$ (which both decrease with $q$), but remains a monotonously increasing function of $q$. The non-monotonic behavior of the collective mode eigenfrequency ${z}_{q}$ found in the analytic continuation through the interval $[2\Delta ,{\omega }_{2}]$ of the real axis^20^ is thus not reflected on the response function. In fact the angular points ${\omega }_{2}$ and ${\omega }_{3}$ only slightly affect the shape of the resonance when they cross it (see in particular the black curve on the left panel of Fig. 4). This is consistent with the finding of ref. ^34^ (see in particular section 4.8 therein): at large $q$, the analytic continuations through windows $[{\omega }_{2},{\omega }_{3}]$ and $[{\omega }_{3},+\,{\rm{\infty }}[$ predict a pole with an eigenfrequency close to that of the Popov-Andrianov branch in window $[2\Delta ,{\omega }_{2}]$. The same robustness towards the choice of the real axis interval through which the analytic continuation is made was noticed by ref. ^21^ for the phononic modes. It is a sign that the Popov-Andrianov collective mode is a fundamental physical phenomenon, which does not depend on a specific configuration of the fermionic continuum.

#### Strong-coupling regime

**Figure 5:**
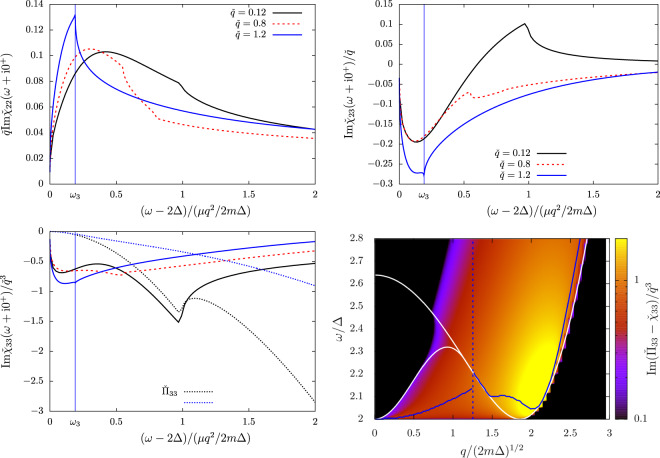
The modulus-modulus (top left panel), modulus-density (top right panel) and density-density (bottom left panel) response functions are shown at unitarity ($\mu /\Delta \simeq 0.86$) in function of the reduced drive frequency $(\omega -2\Delta )/(\mu {q}^{2}/2m\Delta )$ for increasing value of the wavevector $q/\sqrt{2m\Delta }=0.12$ (black solid line), $0.8$ (red dashed line) and $1.2$ (blue solid line). For $q/\sqrt{2m\Delta }=1.2$, we show by a vertical blue line the value of the singularity ${\omega }_{2}={\omega }_{3}\simeq 2.23\Delta$ where the shape of the response functions changes dramatically. In the bottom left panel, we also show the contribution of the pure density-density fluctuations ${\Pi }_{33}$ to the total density response (black and blue dotted line). Bottom right panel: $\text{Im}({\check{\Pi }}_{33}-{\check{\chi }}_{33})$ is shown in colors as a function of $\omega$ and $q$ (the color scale is logarithmic) after division by ${\check{q}}^{3}$. The angular points ${\omega }_{2}$ and ${\omega }_{3}\ge {\omega }_{2}$ are superimposed on the color plot as white solid lines. The global extremum of the function $\omega \mapsto \text{Im}{\chi }_{33}(\omega +i{0}^{+})-\text{Im}{\Pi }_{33}(\omega +i{0}^{+})$ is shown as a blue solid line. Its location is discontinuous in $\check{q}\simeq 1.25$ (vertical dashed line) after which it coincides with the angular point ${\omega }_{3}$ for a range of values of $q$.

Conversely, the increase of $q$ favours the observability of the resonance in both the modulus-density and density-density response functions at strong coupling. On Fig. 5, we show ${\chi }_{23}$ and ${\chi }_{33}$ (as well as ${\chi }_{22}$) at unitarity ($\mu /\Delta \simeq 0.86$) and still at zero temperature. As long as it does not encounter the singularity in ${\omega }_{3}$, a smooth extremum (in ${\chi }_{23}$ and ${\chi }_{33}$ it’s a minimum) whose location increases quadratically with $q$ remains visible. The resonance broadens with $q$, but this is compensated by a deepening of the resonance peak roughly as $q$ in ${\chi }_{23}$ and as ${q}^{3}$ in ${\chi }_{33}$. The resonance in ${\chi }_{33}$ is caused by the order-parameter contribution ${\chi }_{33}-{\Pi }_{33}$ to the density-density fluctuations (compare the blue dotted and the blue solid line on the bottom left panel of Fig. 5), in which it is a global minimum as a function of $\omega$ (rather than a local minimum in ${\chi }_{33}$). To emphasize the dispersion of the resonance, we thus plot on the bottom right panel of Fig. 5, $\text{Im}{\Pi }_{33}-\text{Im}{\chi }_{33}$ divided by ${q}^{3}$ in colors as a function of $\omega$ and $q$. The global extremum of $\omega \mapsto \text{Im}({\Pi }_{33}-{\chi }_{33})$ is shown as a function of $q$ in white solid line. As long as it stays in the window $[2\Delta ,{\omega }_{2}]$, it varies approximatively quadratically with $q$.

Contrarily to what happens at weak-coupling, the resonance shape at strong coupling is much distorted when going through the singularity ${\omega }_{3}$. This effect is particularly visible on the modulus-modulus and modulus-density responses (upper panels of Fig. 5) where the resonance seems broken in ${\omega }_{3}$ such that the smooth extremum has disappeared in favour of a sharp extremum in ${\omega }_{3}$. On the color plot of Fig. 5, the quadratic growth of the resonance frequency is also visibly halted when it encounters the angular point in ${\omega }_{3}$. This is not surprising since the poles found in the analytic continuation through windows $[2\Delta ,{\omega }_{3}]$ and $[{\omega }_{3},+\,\infty [$ are very far apart in this regime^34^. For the value $q/\sqrt{2m\Delta }=1.2$ used in Fig. 5, the analytic continuation through the interval $[2\Delta ,{\omega }_{3}]$ has a pole in ${z}_{q}/\Delta =1.93-0.41i$. In the interval $[{\omega }_{3},+\,\infty ]$, the pole is in ${z}_{q}/\Delta =0.86-0.020i$, with a much lower value of the eigenfrequency and a small damping rate which give this “upper tail” appearance to the response functions at $\omega  > {\omega }_{3}$. Above ${\omega }_{3}$, the behavior of the response functions is in fact similar to what happens in the BEC regime (see below Sec. VI A 3), with a sharp edge pinned at ${\omega }_{3}$ (which becomes the lower edge of the continuum when $q=2\sqrt{2m\mu }$).

#### In the BEC regime

**Figure 6:**
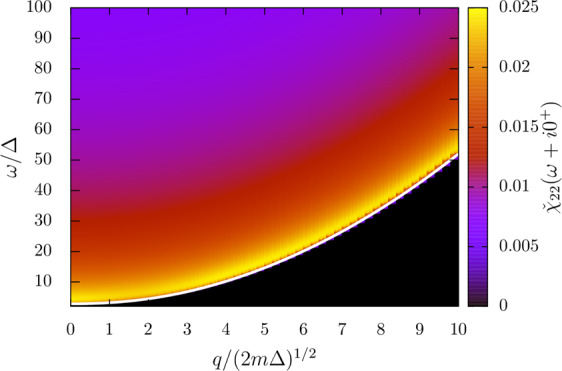
The modulus-modulus response function ${\chi }_{22}$ in the BEC regime ($\mu /\Delta =-\,1$, $\mathrm{1/}{k}_{{\rm{F}}}a\simeq 1.3$) is shown in colors as a function of the wavevector $q$ and drive frequency $\omega$. The lower edge of the continuum ${\omega }_{3}=2\sqrt{{({\hslash }^{2}{q}^{2}/8m+|\mu |)}^{2}+{\Delta }^{2}}$ is shown as a white solid line.

In the BEC regime (that is for us when $\mu  < 0$), the lower-edge of the pair-breaking continuum is no longer flat at low $q$, but increases quadratically with $q$. Although a pole can be found in the analytic continuation through the interval $[{\omega }_{3},+{\rm{\infty }}[$ (the only one available when $\mu  < 0$), its real part always stays below ${\omega }_{3}$, such that no smooth peak appears in the response function. Instead there is only a sharp feature pinned at the lower-edge of the continuum. Figure 6, shows the example of the modulus-modulus response function (the other responses have a similar behavior) at $\mu /\Delta =-\,1$ ($\mathrm{1/}{k}_{F}a\simeq 1.3$). This sharp feature can hardly be interpreted as a collective mode and only reflects the incoherent response of the fermionic continuum when the pairs are tightly bound.

### Near *T*_*c*_

**Figure 7:**
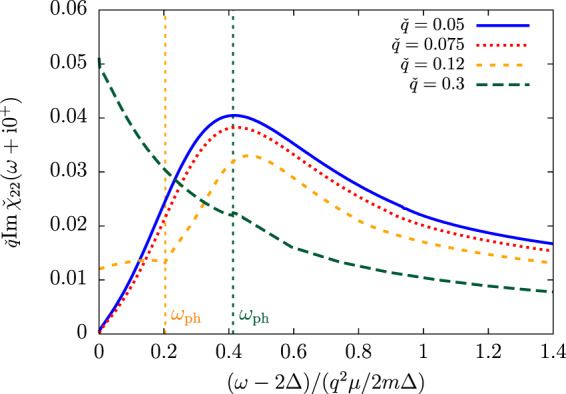
The dispersion of the Popov-Andrianov branch in the modulus-modulus response function near the transition temperature ($\Delta /T=0.1$) and in the weak-coupling regime ${\mu }_{c}/{T}_{{\rm{c}}}\simeq \mu /T=10$ ($\mathrm{1/}{k}_{{\rm{F}}}a\simeq -\,1.15$ with the mean-field equation-of-state). The modulus-modulus response function is plotted as a function of the reduced drive frequency $(\omega -2\Delta )/({q}^{2}\mu /2m\Delta )$ for the values of the wavector $q/\sqrt{2m\Delta }=0.05,0.075,0.12$ and $0.3$ (respectively solid blue, dotted red, dashed orange and long-dashed green lines). The resonance is well visible at low-$q$ but it disappears as the angular point of the quasiparticle-quasihole continuum ${\omega }_{{\rm{ph}}}$ (shown by a vertical dotted line at $(\omega -2\Delta )/({q}^{2}\mu /2m\Delta )=0.204$ and $0.413$ for respectively $q/\sqrt{2m\Delta }=0.12$ and $0.3$) rises with $q$.

At nonzero temperature and even near ${T}_{c}$, we have shown in section V that the Popov-Andrianov resonance exists in the limit $q\to 0$ and is almost insensitive to the quasiparticle-quasihole contributions (38) to the fluctuation matrix $\Pi$. This is no longer the case at higher $q$. The angular point ${\omega }_{{\rm{ph}}}$ of the quasiparticle-quasihole continuum in particular destroys the resonance as it increases (initially linearly) with $q$. This effect is illustrated on Fig. 7 showing the modulus-modulus response function near ${T}_{c}$: for $q/\sqrt{2m\Delta }=0.12$ (orange dashed curve on Fig. 7) the lower tail of the resonance is trimmed by the angular point at ${\omega }_{{\rm{ph}}}$, and for $q/\sqrt{2m\Delta }=0.3$ (long-dashed green curve) it is completely hidden. This can be understood by a simple reasoning: near ${T}_{c}$, ${\omega }_{{\rm{ph}}}$ varies as $q\sqrt{2\mu /m}$ at low $q$^21^, such that it reaches 2Δ for $\check{q}=q/\sqrt{2m\Delta }\approx \sqrt{\Delta /\mu }=O{({T}_{c}-T)}^{1/4}$. The long wavelength limit near ${T}_{c}$ is thus limited to ${q}^{2}/2m\ll {\Delta }^{2}/\mu$ (as in the weak-coupling case at $T=0$ see Eq. (90) in ref. ^34^).

## Conclusion

We have computed the response function matrix of a superfluid Fermi gas in the Random Phase Approximation at nonzero temperature, and used it to study the observability of the order-parameter collective modes. We have shown that the appearance of a resonance inside the pair-breaking continuum associated to the Popov-Andrianov-“Higgs” mode is a very robust phenomenon which concerns not only the modulus-modulus response function but also the modulus-density and density-density responses, which are easier to measure. At weak-coupling the resonance is observable at all values of the wavevector $q$ and is only weakly sensitive to the angular points created in the response functions by the changes of structure of the fermionic continuum. At nonzero temperature, we have shown analytically that the resonance is not destroyed by the presence of excited fermionic quasiparticles, and retains approximatively the same shape as when $T=0$. It also coexists with the low-velocity phononic collective mode which RPA predicts near ${T}_{c}$. The spectral weight of the resonance is enhanced in the modulus-density and density-density responses when $T$ increases, which should favour its observability.

## Appendix A: Derivation of the equations of motion

We give here a few additional steps leading to the equations of motion (22–25). In the particle basis, the equations of motion take the form:

A1 $$\begin{array}{ccc}i\hslash \frac{d{\hat{d}}_{{\bf{k}}}^{{\bf{q}}}}{dt} & = & {\xi }_{{\bf{k}}{\bf{q}}}^{+}{\hat{d}}_{{\bf{k}}}^{{\bf{q}}}-\Delta ({\hat{n}}_{{\bf{k}}}^{{\bf{q}}}+{\hat{\bar{n}}}_{{\bf{k}}}^{{\bf{q}}})+{z}_{{\bf{k}}{\bf{q}}}^{+}({\hat{\Delta }}^{{\bf{q}}}+\phi ({\bf{q}}))\\  &  & +\,{d}_{{\bf{k}}{\bf{q}}}^{+}\frac{{g}_{0}{\hat{n}}_{\uparrow }^{{\bf{q}}}+{g}_{0}{\hat{n}}_{\downarrow }^{{\bf{q}}}+{u}_{+}({\bf{q}})}{2}-{d}_{{\bf{k}}{\bf{q}}}^{-}\frac{{g}_{0}{\hat{n}}_{\uparrow }^{{\bf{q}}}-{g}_{0}{\hat{n}}_{\downarrow }^{{\bf{q}}}-{u}_{-}({\bf{q}})}{2}\end{array}$$

A2 $$\begin{array}{ccc}i\hslash \frac{d{\hat{\bar{d}}}_{{\bf{k}}}^{{\bf{q}}}}{dt} & = & -\,{\xi }_{{\bf{k}}{\bf{q}}}^{+}{\hat{\bar{d}}}_{{\bf{k}}}^{{\bf{q}}}+\Delta ({\hat{n}}_{{\bf{k}}}^{{\bf{q}}}+{\hat{\bar{n}}}_{{\bf{k}}}^{{\bf{q}}})-{z}_{{\bf{k}}{\bf{q}}}^{+}({\hat{\bar{\Delta }}}^{{\bf{q}}}+\bar{\phi }({\bf{q}}))\\  &  & -\,{d}_{{\bf{k}}{\bf{q}}}^{+}\frac{{g}_{0}{\hat{n}}_{\uparrow }^{{\bf{q}}}+{g}_{0}{\hat{n}}_{\downarrow }^{{\bf{q}}}+{u}_{+}({\bf{q}})}{2}-{d}_{{\bf{k}}{\bf{q}}}^{-}\frac{{g}_{0}{\hat{n}}_{\uparrow }^{{\bf{q}}}-{g}_{0}{\hat{n}}_{\downarrow }^{{\bf{q}}}-{u}_{-}({\bf{q}})}{2}\end{array}$$

A3 $$\begin{array}{ccc}i\hslash \frac{d{\hat{n}}_{{\bf{k}}}^{{\bf{q}}}}{dt} & = & -\,{\xi }_{{\bf{k}}{\bf{q}}}^{-}{\hat{n}}_{{\bf{k}}}^{{\bf{q}}}-\Delta ({\hat{d}}_{{\bf{k}}}^{{\bf{q}}}-{\hat{\bar{d}}}_{{\bf{k}}}^{{\bf{q}}})-{z}_{{\bf{k}}{\bf{q}}}^{-}({g}_{0}{\hat{n}}_{\downarrow }^{{\bf{q}}}+{u}_{\uparrow }({\bf{q}}))\\  &  & +\,{d}_{{\bf{k}}{\bf{q}}}^{-}\frac{{\hat{\Delta }}^{{\bf{q}}}+{\hat{\bar{\Delta }}}^{{\bf{q}}}+{\phi }_{+}({\bf{q}})}{2}+{d}_{{\bf{k}}{\bf{q}}}^{+}\frac{{\hat{\Delta }}^{{\bf{q}}}-{\hat{\bar{\Delta }}}^{{\bf{q}}}+{\phi }_{-}({\bf{q}})}{2}\end{array}$$

A4 $$\begin{array}{ccc}i\hslash \frac{d{\hat{\bar{n}}}_{{\bf{k}}}^{{\bf{q}}}}{dt} & = & {\xi }_{{\bf{k}}{\bf{q}}}^{-}{\hat{\bar{n}}}_{{\bf{k}}}^{{\bf{q}}}-\Delta ({\hat{d}}_{{\bf{k}}}^{{\bf{q}}}-{\hat{\bar{d}}}_{{\bf{k}}}^{{\bf{q}}})+{z}_{{\bf{k}}{\bf{q}}}^{-}({g}_{0}{\hat{n}}_{\uparrow }^{{\bf{q}}}+{u}_{\downarrow }({\bf{q}}))\\  &  & -\,{d}_{{\bf{k}}{\bf{q}}}^{-}\frac{{\hat{\Delta }}^{{\bf{q}}}+{\hat{\bar{\Delta }}}^{{\bf{q}}}+{\phi }_{+}({\bf{q}})}{2}+{d}_{{\bf{k}}{\bf{q}}}^{+}\frac{{\hat{\Delta }}^{{\bf{q}}}-{\hat{\bar{\Delta }}}^{{\bf{q}}}+{\phi }_{-}({\bf{q}})}{2},\end{array}$$

where we generalize the notations of refs. ^37,51^ to nonzero temperature:

A5 $$\begin{array}{rcl}{z}_{{\bf{k}}{\bf{q}}}^{\pm }(T) & = & \left(\frac{1}{2}-{\langle {\hat{n}}_{{{\bf{k}}}_{+}}^{{\bf{0}}}\rangle }_{T}\right)\pm \left(\frac{1}{2}-{\langle {\hat{n}}_{{{\bf{k}}}_{-}}^{{\bf{0}}}\rangle }_{T}\right)\\  & = & \frac{{U}_{{{\bf{k}}}_{+}}^{2}-{V}_{{{\bf{k}}}_{+}}^{2}}{2}\mathrm{(1}-2{f}_{{{\bf{k}}}_{+}})\pm \frac{{U}_{{{\bf{k}}}_{-}}^{2}-{V}_{{{\bf{k}}}_{-}}^{2}}{2}\mathrm{(1}-2{f}_{{{\bf{k}}}_{-}})\\  & = & \left[\frac{{\xi }_{{{\bf{k}}}_{+}}}{2{\epsilon }_{{{\bf{k}}}_{+}}}\pm \frac{{\xi }_{{{\bf{k}}}_{-}}}{2{\epsilon }_{{{\bf{k}}}_{-}}}\right]\mathrm{(1}-{f}_{{{\bf{k}}}_{+}}-{f}_{{{\bf{k}}}_{-}})-\left[\frac{{\xi }_{{{\bf{k}}}_{+}}}{2{\epsilon }_{{{\bf{k}}}_{+}}}\mp \frac{{\xi }_{{{\bf{k}}}_{-}}}{2{\epsilon }_{{{\bf{k}}}_{-}}}\right]({f}_{{{\bf{k}}}_{+}}-{f}_{{{\bf{k}}}_{-}})\end{array}$$

A6 $$\begin{array}{ccc}{d}_{{\bf{k}}{\bf{q}}}^{\pm }(T) & = & {\langle {\hat{d}}_{{\bf{k}}+{\bf{q}}\mathrm{/2}}^{{\bf{0}}}\rangle }_{T}\pm {\langle {\hat{d}}_{{\bf{k}}-{\bf{q}}\mathrm{/2}}^{{\bf{0}}}\rangle }_{T}=-[{U}_{{{\bf{k}}}_{+}}{V}_{{{\bf{k}}}_{+}}\mathrm{(1}-2{f}_{{{\bf{k}}}_{+}})\pm {U}_{{{\bf{k}}}_{-}}{V}_{{{\bf{k}}}_{-}}\mathrm{(1}-2{f}_{{{\bf{k}}}_{-}})]\\  & = & -\left[\frac{\Delta }{2{\epsilon }_{{{\bf{k}}}_{+}}}\pm \frac{\Delta }{2{\epsilon }_{{{\bf{k}}}_{-}}}\right]\mathrm{(1}-{f}_{{{\bf{k}}}_{+}}-{f}_{{{\bf{k}}}_{-}})+\left[\frac{\Delta }{2{\epsilon }_{{{\bf{k}}}_{+}}}\mp \frac{\Delta }{2{\epsilon }_{{{\bf{k}}}_{-}}}\right]({f}_{{{\bf{k}}}_{+}}-{f}_{{{\bf{k}}}_{-}}\mathrm{)}.\end{array}$$

Adding and subtracting Eqs. (A1) to (A2) and Eqs. (A3) to (A4) and performing the change of basis (21) (one can use the explicit relations given in Appendix C of ref. ^51^) yields the equations of motion (22–25) in the quasiparticle basis. Rederiving with respect to time yields:

A7 $$\begin{array}{rcl}-\,{({\epsilon }_{{\bf{k}}{\bf{q}}}^{+})}^{2}{\hat{y}}_{{\bf{k}}}^{{\bf{q}}}-\frac{{d}^{2}{\hat{y}}_{{\bf{k}}}^{{\bf{q}}}}{d{t}^{2}} & = & \mathrm{(1}-{f}_{{{\bf{k}}}_{+}}-{f}_{{{\bf{k}}}_{-}})[{W}_{{\bf{k}}{\bf{q}}}^{-}i\hslash {\partial }_{t}(\delta {\hat{\Delta }}^{{\bf{q}}}+\delta {\hat{\bar{\Delta }}}^{{\bf{q}}}+{\phi }_{+}({\bf{q}}))\\  &  & -\,{w}_{{\bf{k}}{\bf{q}}}^{+}i\hslash {\partial }_{t}({g}_{0}[\delta {\hat{n}}_{\uparrow }^{{\bf{q}}}+\delta {\hat{n}}_{\downarrow }^{{\bf{q}}}]+{u}_{+}({\bf{q}}))\\  &  & +\,{\epsilon }_{{\bf{k}}{\bf{q}}}^{+}{W}_{{\bf{k}}{\bf{q}}}^{+}({\hat{\Delta }}^{{\bf{q}}}-{\hat{\bar{\Delta }}}^{{\bf{q}}}+{\phi }_{-}({\bf{q}}))\\  &  & -{\epsilon }_{{\bf{k}}{\bf{q}}}^{+}{w}_{{\bf{k}}{\bf{q}}}^{-}({g}_{0}[{\hat{n}}_{\uparrow }^{{\bf{q}}}-{\hat{n}}_{\downarrow }^{{\bf{q}}}]-{u}_{-}({\bf{q}}))]\end{array}$$

A8 $$\begin{array}{rcl}-\,{({\epsilon }_{{\bf{k}}{\bf{q}}}^{-})}^{2}{\hat{h}}_{{\bf{k}}}^{{\bf{q}}}-\frac{{d}^{2}{\hat{h}}_{{\bf{k}}}^{{\bf{q}}}}{d{t}^{2}} & = & ({f}_{{{\bf{k}}}_{+}}-{f}_{{{\bf{k}}}_{-}})[{w}_{{\bf{k}}{\bf{q}}}^{+}i\hslash {\partial }_{t}(\delta {\hat{\Delta }}^{{\bf{q}}}+\delta {\hat{\bar{\Delta }}}^{{\bf{q}}}+{\phi }_{+}({\bf{q}}))\\  &  & +\,{W}_{{\bf{k}}{\bf{q}}}^{-}i\hslash {\partial }_{t}({g}_{0}[\delta {\hat{n}}_{\uparrow }^{{\bf{q}}}+\delta {\hat{n}}_{\downarrow }^{{\bf{q}}}]+{u}_{+}({\bf{q}}))\\  &  & +\,{\epsilon }_{{\bf{k}}{\bf{q}}}^{-}{w}_{{\bf{k}}{\bf{q}}}^{-}({\hat{\Delta }}^{{\bf{q}}}-{\hat{\bar{\Delta }}}^{{\bf{q}}}+{\phi }_{-}({\bf{q}}))\\  &  & +\,{\epsilon }_{{\bf{k}}{\bf{q}}}^{-}{W}_{{\bf{k}}{\bf{q}}}^{+}({g}_{0}[{\hat{n}}_{\uparrow }^{{\bf{q}}}-{\hat{n}}_{\downarrow }^{{\bf{q}}}]-{u}_{-}({\bf{q}}))]\end{array}$$

A9 $$\begin{array}{rcl}-\,{({\epsilon }_{{\bf{k}}{\bf{q}}}^{+})}^{2}{\hat{s}}_{{\bf{k}}}^{{\bf{q}}}-\frac{{d}^{2}{\hat{s}}_{{\bf{k}}}^{{\bf{q}}}}{d{t}^{2}} & = & \mathrm{(1}-{f}_{{{\bf{k}}}_{+}}-{f}_{{{\bf{k}}}_{-}})[{W}_{{\bf{k}}{\bf{q}}}^{+}i\hslash {\partial }_{t}({\hat{\Delta }}^{{\bf{q}}}-{\hat{\bar{\Delta }}}^{{\bf{q}}}+{\phi }_{-}({\bf{q}}))\\  &  & -\,{w}_{{\bf{k}}{\bf{q}}}^{-}i\hslash {\partial }_{t}({g}_{0}[{\hat{n}}_{\uparrow }^{{\bf{q}}}-{\hat{n}}_{\downarrow }^{{\bf{q}}}]-{u}_{-}({\bf{q}}))\\  &  & +\,{\epsilon }_{{\bf{k}}{\bf{q}}}^{+}{W}_{{\bf{k}}{\bf{q}}}^{-}(\delta {\hat{\Delta }}^{{\bf{q}}}+\delta {\hat{\bar{\Delta }}}^{{\bf{q}}}+{\phi }_{+}({\bf{q}}))\\  &  & -\,{\epsilon }_{{\bf{k}}{\bf{q}}}^{+}{w}_{{\bf{k}}{\bf{q}}}^{+}({g}_{0}[\delta {\hat{n}}_{\uparrow }^{{\bf{q}}}+\delta {\hat{n}}_{\downarrow }^{{\bf{q}}}]+{u}_{+}({\bf{q}}))]\end{array}$$

A10 $$\begin{array}{rcl}-\,{({\epsilon }_{{\bf{k}}{\bf{q}}}^{-})}^{2}{\hat{m}}_{{\bf{k}}}^{{\bf{q}}}-\frac{{d}^{2}{\hat{m}}_{{\bf{k}}}^{{\bf{q}}}}{d{t}^{2}} & = & -\,(\,{f}_{{{\bf{k}}}_{+}}-{f}_{{{\bf{k}}}_{-}})[{w}_{{\bf{k}}{\bf{q}}}^{-}i\hslash {\partial }_{t}({\hat{\Delta }}^{{\bf{q}}}-{\hat{\bar{\Delta }}}^{{\bf{q}}}+{\phi }_{-}({\bf{q}}))\\  &  & +\,{W}_{{\bf{k}}{\bf{q}}}^{+}i\hslash {\partial }_{t}({g}_{0}[{\hat{n}}_{\uparrow }^{{\bf{q}}}-{\hat{n}}_{\downarrow }^{{\bf{q}}}]-{u}_{-}({\bf{q}}))\\  &  & +\,{\epsilon }_{{\bf{k}}{\bf{q}}}^{-}{w}_{{\bf{k}}{\bf{q}}}^{+}(\delta {\hat{\Delta }}^{{\bf{q}}}+\delta {\hat{\bar{\Delta }}}^{{\bf{q}}}+{\phi }_{+}({\bf{q}}))\\  &  & +\,{\epsilon }_{{\bf{k}}{\bf{q}}}^{-}{W}_{{\bf{k}}{\bf{q}}}^{-}({g}_{0}[\delta {\hat{n}}_{\uparrow }^{{\bf{q}}}+\delta {\hat{n}}_{\downarrow }^{{\bf{q}}}]+{u}_{+}({\bf{q}}))]\mathrm{}.\end{array}$$

We resum this system to form the collective quantities (26–29) and derive the 4 × 4 linear system (30).

## Appendix B: Numerical calculation of the response functions

To numerically compute the fluctuation matrix $\Pi$, we first compute its spectral density:

B1 $${\rm{I}}{\rm{m}}\,{\check{\Pi }}_{ij}(\omega +i{0}^{+})=-\pi ({\rho }_{ij}^{(pp)}(\omega )+{\rho }_{ij}^{(ph)}(\omega )),$$

where ${\rho }_{ij}^{(pp)}$ and ${\rho }_{ij}^{(ph)}(\omega )$ are respectively the contributions of the quasiparticle-quasiparticle integral $\Sigma$ and quasparticle-quasihole integral $S$ to the spectral density of ${\Pi }_{ij}$. Denoting $u={\bf{k}}\cdot {\bf{q}}/kq$, and restricting, without loss of generality, to $\omega  > 0$, we have, explicitly:

B2 $${\rho }_{ij}^{(pp)}(\omega )=\frac{2\pi \Delta }{{k}_{\Delta }^{3}}{\eta }_{ij}\,{\int }_{0}^{+{\rm{\infty }}}\,{k}^{2}dk\,{\int }_{0}^{1}\,du{a}_{i,{\bf{k}}{\bf{q}}}{a}_{j,{\bf{k}}{\bf{q}}}(1-{f}_{{\bf{k}}+{\bf{q}}/2}-{f}_{{\bf{k}}-{\bf{q}}/2})\delta (\omega -{\epsilon }_{{\bf{k}}{\bf{q}}}^{+}),$$

We have introduced ${k}_{\Delta }=\sqrt{2m\Delta }$, the coefficients ${a}_{\mathrm{1,}{\bf{k}}{\bf{q}}}={W}_{{\bf{k}},{\bf{q}}}^{+}$, ${a}_{\mathrm{2,}{\bf{k}}{\bf{q}}}={W}_{{\bf{k}},{\bf{q}}}^{-}$ and ${a}_{\mathrm{3,}{\bf{k}}{\bf{q}}}={w}_{{\bf{k}},{\bf{q}}}^{+}$ and the signs ${\eta }_{ij}$, read from (36)

B3 $$\eta =(\begin{array}{ccc}1 & 1 & -\,1\\ 1 & 1 & -\,1\\ -\,1 & -\,1 & 1\end{array}).$$

For the particle-hole contribution, we have

B4 $${\rho }_{ij}^{(ph)}(\omega )=-\frac{2\pi \Delta }{{k}_{\Delta }^{3}}\,{\int }_{0}^{+{\rm{\infty }}}\,{k}^{2}dk\,{\int }_{0}^{1}\,du{b}_{i,{\bf{k}}{\bf{q}}}{b}_{j,{\bf{k}}{\bf{q}}}({f}_{{\bf{k}}+{\bf{q}}/2}-{f}_{{\bf{k}}-{\bf{q}}/2})[\delta (\omega -{\epsilon }_{{\bf{k}}{\bf{q}}}^{-})-{\sigma }_{ij}\delta (\omega +{\epsilon }_{{\bf{k}}{\bf{q}}}^{-})].$$

Here, ${b}_{1,{\bf{k}}{\bf{q}}}={w}_{{\bf{k}},{\bf{q}}}^{-}$, ${b}_{\mathrm{2,}{\bf{k}}{\bf{q}}}={w}_{{\bf{k}},{\bf{q}}}^{+}$ and ${b}_{\mathrm{3,}{\bf{k}}{\bf{q}}}={W}_{{\bf{k}},{\bf{q}}}^{-}$ and the sign ${\sigma }_{ij}$ is +1 for ${S}^{\epsilon }$ matrix elements and −1 for the ${S}^{\omega }$:

B5 $$\sigma =(\begin{array}{ccc}1 & -\,1 & -\,1\\ -\,1 & 1 & 1\\ -\,1 & 1 & 1\end{array}).$$

In (B2) and (B4), we have used the symmetry or antisymmetry of the coefficients ${a}_{i,{\bf{k}}{\bf{q}}}{a}_{j,{\bf{k}}{\bf{q}}}$ and ${b}_{i,{\bf{k}}{\bf{q}}}{b}_{j,{\bf{k}}{\bf{q}}}$ with respect to the exchange $u\leftrightarrow -\,u$ to restrict the integral to $u > 0$.

In the quasiparticle-quasiparticle spectral density (B2), we give the resonance angle:

B6 $${u}_{r}=\frac{m\omega }{kq}{\left(\frac{{\xi }^{2}-({\omega }^{2}-4{\Delta }^{2}\mathrm{)/4}}{{\xi }^{2}-{\omega }^{2}\mathrm{/4}}\right)}^{\mathrm{1/2}}\,{\rm{with}}\,\xi ={k}^{2}\mathrm{/2}m+{q}^{2}\mathrm{/8}m-\mu $$

For ${\omega }_{1} < \omega  < {\omega }_{2}$, this quantity is comprised in $[0,1]$ (such that the resonance in (B2) is reached) for $k\in [{k}_{1},{k}_{2}]$ with ${k}_{1}$ and ${k}_{2}$ solutions of ${({\epsilon }_{{\bf{k}}+{\bf{q}}/2}+{\epsilon }_{{\bf{k}}-{\bf{q}}/2})|}_{u=0}=\omega$. For ${\omega }_{2} < \omega  < {\omega }_{3}$ the resonance is reached for $k\in [{k}_{1},{k{\prime} }_{1}]$ and $k\in [{k{\prime} }_{2},{k}_{2}]$ with ${k{\prime} }_{1}$ and ${k{\prime} }_{2}$ solutions of ${\epsilon }_{k+q/2}+{\epsilon }_{k-q/2}=\omega$. Finally for $\omega  > {\omega }_{3}$ the resonance is reached for $k\in [{k{\prime} }_{2},{k}_{2}]$ only. Using the variable $y=2\xi /\omega$ instead of the wavenumber $k$, and $t={\rm{argch}}\,(\omega /2\Delta )$ instead of the drive frequency, then using the Dirac delta to integrate analytically over the scattering angle *u*, we have

B7 $$\begin{array}{rcl}{\rho }_{ij}^{({\rm{pp}})}(\omega ) & = &\frac{\pi }{4{\tilde{q}}\,{\rm{ch}}\,t}\\  &  & \times\,\left\{\begin{array}{l}\int_{-{\rm{th}}t}^{{\rm{th}}t}dy\frac{{\tilde{W}}_{ij}(y)(1-{\tilde{f}}_{+}(y)-{\tilde{f}}_{-}(y))}{\sqrt{({{\rm{th}}}^{2}t-{y}^{2})(1-{y}^{2})}}\quad{\rm{if}}\,{\omega}_{1} < \omega  < {\omega }_{2}\\ \left[\int_{-{\rm{th}}t}^{{y}_{1}{\prime} }dy+\int_{{y}_{2}{\prime}}^{{\rm{th}}t}dy\right]\frac{{\tilde{W}}_{ij}(y)(1-{\tilde{f}}_{+}(y)-{\tilde{f}}_{-}(y))}{\sqrt{({{\rm{th}}}^{2}t-{y}^{2})(1-{y}^{2})}}\quad{\rm{if}}\,{\omega}_{2} < \omega  < {\omega }_{3}\\ \int_{{y}_{2}{\prime}}^{{\rm{th}}t}\frac{{\tilde{W}}_{ij}(y)(1-{\tilde{f}}_{+}(y)-{\tilde{f}}_{-}(y))}{\sqrt{({{\rm{th}}}^{2}t-{y}^{2})(1-{y}^{2})}}\quad{\rm{if}}\,\omega > {\omega }_{3}\end{array}\right.\end{array}$$

where ${y{\prime} }_{1}$ and ${y{\prime} }_{2}$ are deduced from ${k{\prime} }_{1}$ and ${k{\prime} }_{2}$ by the change of variable given above, and the functions *W*_*ij*_ are:

B8 $${\tilde{W}}_{11}(y)=\frac{2}{1-{y}^{2}}$$

B9 $${\tilde{W}}_{22}(y)=\frac{2{y}^{2}}{1-{y}^{2}}$$

B10 $${\tilde{W}}_{12}(y)=\frac{2y}{1-{y}^{2}}$$

B11 $${\tilde{W}}_{13}(y)=2\,{\rm{ch}}\,t$$

B12 $${\tilde{W}}_{23}(y)=2y\,{\rm{ch}}\,t$$

B13 $${\tilde{W}}_{33}(y)=2\,{{\rm{ch}}}^{2}\,t(1-{y}^{2}).$$

In our integration variables, the Fermi-Dirac occupation numbers have the expression

B14 $${\tilde{f}}_{\pm }(y)=1/(1+\exp [\sqrt{{{\rm{ch}}}^{2}\,t{(y\pm kq{u}_{r}/m\omega )}^{2}+1}\times \Delta /T]).$$

In the quasiparticle-quasihole spectral density (B4), we give the resonance angle expressed in terms of $\xi ={k}^{2}/2m+{q}^{2}/8m-\mu$ has the expression (B6) given above. Whatever the value of $\omega$ this angle exists (*i*.*e*. ${u}_{r}\in [0,1]$) for $k\in [{\tilde{k}}_{1},\infty [$, with ${\tilde{k}}_{1}$ the solution of ${\epsilon }_{k+q/2}-{\epsilon }_{k-q/2}=\omega$. When $\omega  < {\omega }_{{\rm{ph}}}$, it also exists for $k\in [{\tilde{k}}_{3},{\tilde{k}}_{2}]$, with ${\tilde{k}}_{3},{\tilde{k}}_{2}$ the two solutions of ${\epsilon }_{k+q/2}-{\epsilon }_{k-q/2}=-\,\omega$. Using the variable $y=\omega /2\xi$ instead of the wavenumber $k$, and $t=\arccos \,(\omega /2\Delta )$ instead of the drive frequency, then using the Dirac delta to integrate analytically over the scattering angle $u$, we have:

B15 $${\rho }_{ij}^{({\rm{ph}})}(\omega )=-\,\frac{\pi }{4\tilde{q}\,\cos \,t}[{\int }_{0}^{{\tilde{y}}_{1}}\,dy-\Theta ({\omega }_{{\rm{ph}}}-\omega )\,{\int }_{{\tilde{y}}_{3}}^{{\tilde{y}}_{2}}\,dy]\frac{{\tilde{w}}_{ij}(y)({\tilde{f}}_{+}(y)-{\tilde{f}}_{-}(y))}{\sqrt{(1+{y}^{2}\,{\tan }^{2}\,t)(1-{y}^{2})}},$$

where ${\tilde{y}}_{i}$ is related to ${\tilde{k}}_{i}$ by the change of variable given above, and the functions ${w}_{ij}$ are

B16 $${\tilde{w}}_{11}(y)=\frac{2{y}^{2}}{1-{y}^{2}}$$

B17 $${\tilde{w}}_{22}(y)=\frac{2}{1-{y}^{2}}$$

B18 $${\tilde{w}}_{12}(y)=\frac{2y}{1-{y}^{2}}$$

B19 $${\tilde{w}}_{13}(y)=2\,\cos \,t$$

B20 $${\tilde{w}}_{23}(y)=\frac{2\,{\rm{\cos }}\,t}{y}$$

B21 $${\tilde{w}}_{33}(y)=2\,{\cos }^{2}\,t\frac{1-{y}^{2}}{{y}^{2}}.$$

Here, the Fermi-Dirac occupation numbers have the expression

B22 $${\tilde{f}}_{\pm }(y)=1/(1+\exp [\sqrt{{\cos }^{2}\,t{(1/y\pm kq{u}_{r}/m\omega )}^{2}+1}\times \Delta /T]).$$

Finally, to compute the full function, we use the spectral density to integrate over energies:

B23 $${\check{\Pi }}_{ij}({\omega }_{0})={\int }_{0}^{+{\rm{\infty }}}\,d\omega \left[\frac{{\rho }_{ij}(\omega )}{{\omega }_{0}-\omega },-,{\sigma }_{ij},\frac{{\rho }_{ij}(\omega )}{{\omega }_{0}+\omega },+,(,{\delta }_{i1},{\delta }_{j1},+,{\delta }_{i2},{\delta }_{j2},),\frac{\pi \sqrt{\omega }}{\sqrt{8(1+{(\omega /2-\mu /\Delta )}^{2})}}\right].$$

In $${\check{\Pi }}_{11}$$ and $${\check{\Pi }}_{22}$$, the divergence at large $$\omega $$ is regularized by the counter-term $$4\pi \,{\int }_{0}^{\infty }\,\frac{{k}^{2}dk}{{k}_{\Delta }^{3}}\frac{\Delta }{2{\epsilon }_{k}}=$$$${\int }_{0}^{+\infty }\,d\omega \frac{\pi \sqrt{\omega }}{8(1+{(\omega /2-\mu /\Delta )}^{2})}$$.
